# Identification of immunological characterization and Anoikis-related molecular clusters in rheumatoid arthritis

**DOI:** 10.3389/fmolb.2023.1202371

**Published:** 2023-11-17

**Authors:** Jianan Zhao, Kai Wei, Yiming Shi, Ping Jiang, Lingxia Xu, Cen Chang, Linshuai Xu, Yixin Zheng, Yu Shan, Jia Liu, Li Li, Shicheng Guo, Steven J. Schrodi, Rongsheng Wang, Dongyi He

**Affiliations:** _1_ Department of Rheumatology, Shanghai Guanghua Hospital, Shanghai University of Traditional Chinese Medicine, Shanghai, China; ^2^ Guanghua Clinical Medical College, Shanghai University of Traditional Chinese Medicine, Shanghai, China; ^3^ Institute of Arthritis Research in Integrative Medicine, Shanghai Academy of Traditional Chinese Medicine, Shanghai, China; ^4^ Arthritis Institute of Integrated Traditional and Western Medicine, Shanghai Chinese Medicine Research Institute, Shanghai, China; ^5^ Computation and Informatics in Biology and Medicine, University of Wisconsin-Madison, Madison, WI United States; ^6^ Department of Medical Genetics, School of Medicine and Public Health, University of Wisconsin-Madison, Madison, WI, United States

**Keywords:** rheumatoid arthritis, anoikis-related molecular clusters, anoikis, cell death, immunological characterization

## Abstract

**Objective:** To investigate the potential association between Anoikis-related genes, which are responsible for preventing abnormal cellular proliferation, and rheumatoid arthritis (RA).

**Methods:** Datasets GSE89408, GSE198520, and GSE97165 were obtained from the GEO with 282 RA patients and 28 healthy controls. We performed differential analysis of all genes and *HLA* genes. We performed a protein-protein interaction network analysis and identified hub genes based on *STRING* and cytoscape. Consistent clustering was performed with subgrouping of the disease. SsGSEA were used to calculate immune cell infiltration. Spearman’s correlation analysis was employed to identify correlations. Enrichment scores of the GO and KEGG were calculated with the ssGSEA algorithm. The WGCNA and the *DGIdb* database were used to mine hub genes’ interactions with drugs.

**Results:** There were 26 differentially expressed Anoikis-related genes (*FDR* = 0.05, log2FC = 1) and HLA genes exhibited differential expression (*P* < 0.05) between the disease and control groups. Protein-protein interaction was observed among differentially expressed genes, and the correlation between *PIM2* and *RAC2* was found to be the highest; There were significant differences in the degree of immune cell infiltration between most of the immune cell types in the disease group and normal controls (*P* < 0.05). Anoikis-related genes were highly correlated with HLA genes. Based on the expression of Anoikis-related genes, RA patients were divided into two disease subtypes (cluster1 and cluster2). There were 59 differentially expressed Anoikis-related genes found, which exhibited significant differences in functional enrichment, immune cell infiltration degree, and *HLA* gene expression (*P* < 0.05). Cluster2 had significantly higher levels in all aspects than cluster1 did. The co-expression network analysis showed that cluster1 had 51 hub differentially expressed genes and cluster2 had 72 hub differentially expressed genes. Among them, three hub genes of cluster1 were interconnected with 187 drugs, and five hub genes of cluster2 were interconnected with 57 drugs.

**Conclusion:** Our study identified a link between Anoikis-related genes and RA, and two distinct subtypes of RA were determined based on Anoikis-related gene expression. Notably, cluster2 may represent a more severe state of RA.

## 1 Introduction

Rheumatoid arthritis (RA) is a chronic autoimmune disease that triggers inflammation in the joints, leading to potential long-term joint damage and disability. Notably, RA can also extend beyond the joints to affect vital organs such as the lungs, heart, blood vessels, skin, and eyes. It is estimated that approximately 0.5% of the adult population worldwide are affected by RA, with a higher incidence rate observed in women compared to men. While individuals of all ages can be affected by this condition, the peak age of onset is typically between 50 and 59 years (Smith and Berman, 2022). The current therapeutic interventions for RA consist of disease-modifying antirheumatic drugs (DMARDs), nonsteroidal anti-inflammatory drugs (NSAIDs), and biologics. While analgesics and NSAIDs can alleviate pain and stiffness associated with RA, their efficacy is often limited, and NSAIDs may involve the risk of gastrointestinal and cardiac toxicity (Zhao et al., 2022a). Although DMARDs, which constitute the primary treatment for RA, can be administered in combination, their multiple adverse effects include hepatotoxicity, hematometabolic disorders, nausea, and interstitial lung disease. Biological agents such as anti-tumor necrosis factor (TNF)-α antibodies have demonstrated efficacy in treating RA; however, their clinical use carries the risk of infusion and injection site infections, and their efficacy may vary depending on the patient’s individual needs (Zhao et al., 2022a). The introduction of these novel therapies has improved the clinical management of RA patients (Zhao et al., 2022a). Nevertheless, due to the complex and heterogeneous nature of the pathogenesis of RA, a substantial portion of patients exhibit an inadequate clinical response, highlighting the need for targeted development of innovative therapeutics.

The term “Anoikis” was first introduced in 1990 (Frisch and Francis, 1994). It is a crucial mechanism for preventing the continued growth of developmentally abnormal cells or attachment to inappropriate matrix when there is no interaction with the extracellular matrix (Taddei et al., 2012). The loss of extracellular matrix attachment disrupts integrin connections, leading to rapid endothelial cell death (Meredith et al., 1993). Anoikis mainly occurs through two pathways: intrinsic and extrinsic pathways. Intrinsic pathway involves mitochondria as a critical organelle, and the key events are mitochondrial permeabilization and regulation of Bcl-2 protein family. The extrinsic pathway involves cell surface death receptor proteins, such as Fas or TNFR, which gradually forms a death-inducing signaling complex and activate downstream caspase 8, eventually leading to Anoikis (Gilmore, 2005). Tumor cells are considered an essential condition in the tumor metastasis process due to their insensitivity to Anoikis, which is called Anoikis resistance. The current understanding suggests that the primary mechanisms behind Anoikis resistance include alterations in integrin expression patterns, excessive expression of extracellular matrix, activation of survival signals induced by oxidative stress, hypoxic microenvironments, and expression of key molecules such as Twist, HGF/Met, EphA2 receptors, and TrkB (Taddei et al., 2012).

There are multiple types of cells in RA that collectively contribute to the abnormal pathological features of RA. RA FLS exhibit multiple tumor-like characteristics and survive and proliferate excessively in tumor-like microenvironments. The abnormal proliferation of RA FLS is partially attributed to the inhibition of cell apoptosis (Bartok and Firestein, 2010; Bottini and Firestein, 2013). RA FLS exhibits characteristics of invasive growth and has been observed in experiments to not rely on wall attachment for growth (Lafyatis et al., 1989). Studies have reported that RA FLS increases its resistance to Anoikis and promotes abnormal pathological characteristics through upregulation of *CTFG* mediated by *ADAM15*/*YAP1* (Janczi et al., 2023). Additionally, the hypoxic microenvironment in RA joints is also an important cause of Anoikis resistance in RA FLS (Taddei et al., 2012; Zhao et al., 2022b). The relationship between other immune cells in RA and Anoikis resistance remains unclear, therefore in this article, we aim to provide theoretical references for the development of clinical diagnosis and treatment plans by analyzing the potential connection between Anoikis -related genes and RA.

## 2 Materials and methods

### 2.1 Data source and processing

The GSE89408, GSE198520, and GSE97165 datasets were downloaded from the GEO database (GEO Accession viewer (nih.gov)). The samples from GSE89408, GSE198520, and GSE97165 are all derived from synovial biopsy tissue samples obtained from individuals with or without RA. Raw counts data from the downloaded datasets were converted into fpkm data and log2 (fpkm+1) was applied, as shown in Table 1. For annotation, the downloaded GEO dataset was annotated based on the GENCODE (V38) gtf annotation file, and coding genes were extracted. Probe IDs were converted to gene symbols, duplicates were removed, and batch effect was removed before merging the data. Subsequent analyses were based on the merged data. An anokis-related gene set was selected from the MSigDB (V7.4) database (GSEA | MSigDB (gsea-msigdb.org)). The complete analysis workflow is shown in Supplementary Figure S1.

**TABLE 1:** Sample information.

	Rheumatoid arthritis	Normal	Data processing	Follow-up processing
GSE198520	92	0	Counts to fpkm	log2 (fpkm+1)
GSE89408	152	28	Counts to fpkm	log2 (fpkm+1)
GSE97165	38	0	Counts to fpkm	log2 (fpkm+1)

### 2.2 Gene difference analysis

To identify differentially expressed genes between disease and control groups, the expression profile data from the merged dataset and the disease/control groupings were used. Differential analysis on all genes was performed using the R package limma, and volcano plots, and heat maps were generated for Anoikis-related genes. Limma is based on a linear model and employs weighted least squares to estimate differential gene expression. It corrects for multiple testing issues using Bayesian methods. Genes were considered downregulated if the false discovery rate (*FDR*) < 0.05 and log2FC < 1, and upregulated if the *FDR* <0.05 and log2FC > 1. The R package RCircos was used to generate a chromosome position diagram of differentially expressed Anoikis-related genes to determine their positions on the chromosome. The gene re-annotation file was downloaded from GENCODE (https://ftp.ebi.ac.uk/pub/databases/gencode/Gencode_human/release_38/gencode.v38.annotation.gff3.gz), which provided information for all differentially expressed genes. The String database was used to construct a PPI network based on differentially expressed Anoikis-related genes. Spearman correlation analysis was performed on the differentially expressed Anoikis-related genes between two scenarios: all samples and disease samples. Using Spearman’s correlation analysis, we evaluated the correlation between differentially expressed Anoikis-related genes across all and disease samples. When *p* < 0.05 is obtained, a statistically significant correlation between the two variables is recognized. Additionally, we analyzed the differential expression of *HLA* genes between the disease and control groups, along with the Spearman correlation analysis between Anoikis-related genes and *HLA* genes.

### 2.3 Analysis of immune infiltration

We used ssGSEA to quantify immune cell infiltration. Immune response gene set enrichment scores were calculated and analyzed for differences between subgroups with immune response gene sets obtained from the immport database (https://www.immport.org/shared/genelists), consisting of 17 immune response gene sets. Additionally, we evaluated the Spearman correlation between Anoikis-related genes and immune response gene set enrichment scores.

### 2.4 Consistency clustering and disease subtyping

We performed consensus clustering using the R package ConsensusClusterPlus based on Anoikis-related differentially expressed genes and disease sample data to identify molecular subtypes based on the optimal clustering K value. The distance metric used for clustering was km, and the clustering method was euclidean; 1,000 repetitions were performed to ensure the stability of the classification. We performed PCA clustering analysis on the disease expression data to show the aggregation status between different subgroups. Based on the subtypes, we analyzed the expression differences of Anoikis-related genes between subtypes and generated heat maps and box plots to visualize the differential expression of Anoikis-related genes.

### 2.5 Functional enrichment differences

We calculated the enrichment scores of GO and KEGG pathways using the ssGSEA algorithm from the R package GSVA. We analyzed the enrichment differences of GO and KEGG pathways among the groups and plotted heatmaps.

### 2.6 WGCNA analysis

WGCNA analysis was performed using the R package WGCNA. First, a similarity matrix was constructed based on the gene expression data. The gene expression similarity matrix was then transformed into an adjacency matrix, with β as the soft threshold, and the network type was signed. The adjacency matrix was then transformed into a topological overlap measure (TOM) matrix, which described the degree of association between genes. The module membership (MM), which measured the identity of a gene in a module, was evaluated based on the Pearson correlation between the gene’s expression profile across all samples and the expression profile of the feature vector gene ME. Finally, gene significance (GS) was calculated to measure the correlation between genes and external information. We identified differentially expressed genes between subtypes and intersected them with hub genes selected by WGCNA to construct a PPI network. Hub gene enrichment in different clusters through further GO and KEGG analysis, with a significance threshold set at *FDR* <0.05 to determine statistically significant enrichment results. Subsequently, we validated differential gene expression across different clusters using RNA-seq expression data from clinical synovial tissue samples recruited from the Guanghua Hospital Precision Medicine Research Cohort, including 9 RA patients and 15 osteoarthritis (OA) patients (Zhang et al., 2022). Hub nodes in the PPI network were identified using cytoscape, and gene degree was used to filter hub genes. Genes with high degree of association were considered hub key genes. We then used the DGIdb database v4.2.0 (https://www.dgidb.org/) to query for drug interactions with these hub genes and presented the interaction relationships using a Sankey diagram.

### 2.7 Statistical analysis

For differential significance analysis, Wilcoxon test was used for comparisons between two groups unless otherwise specified, and Kruskal–Wallis test was used for comparisons between more than two groups. R version 4.1.2 was used for statistical analysis. In the figures, ns indicates *P* > 0.05, * indicates *P* < 0.05, **P < 0.01, ***P < 0.001, and **** indicates *P* < 0.0001.

## 3 Results

### 3.1 Landscape of anoikis-related genes in the disease

**FIGURE 1:**
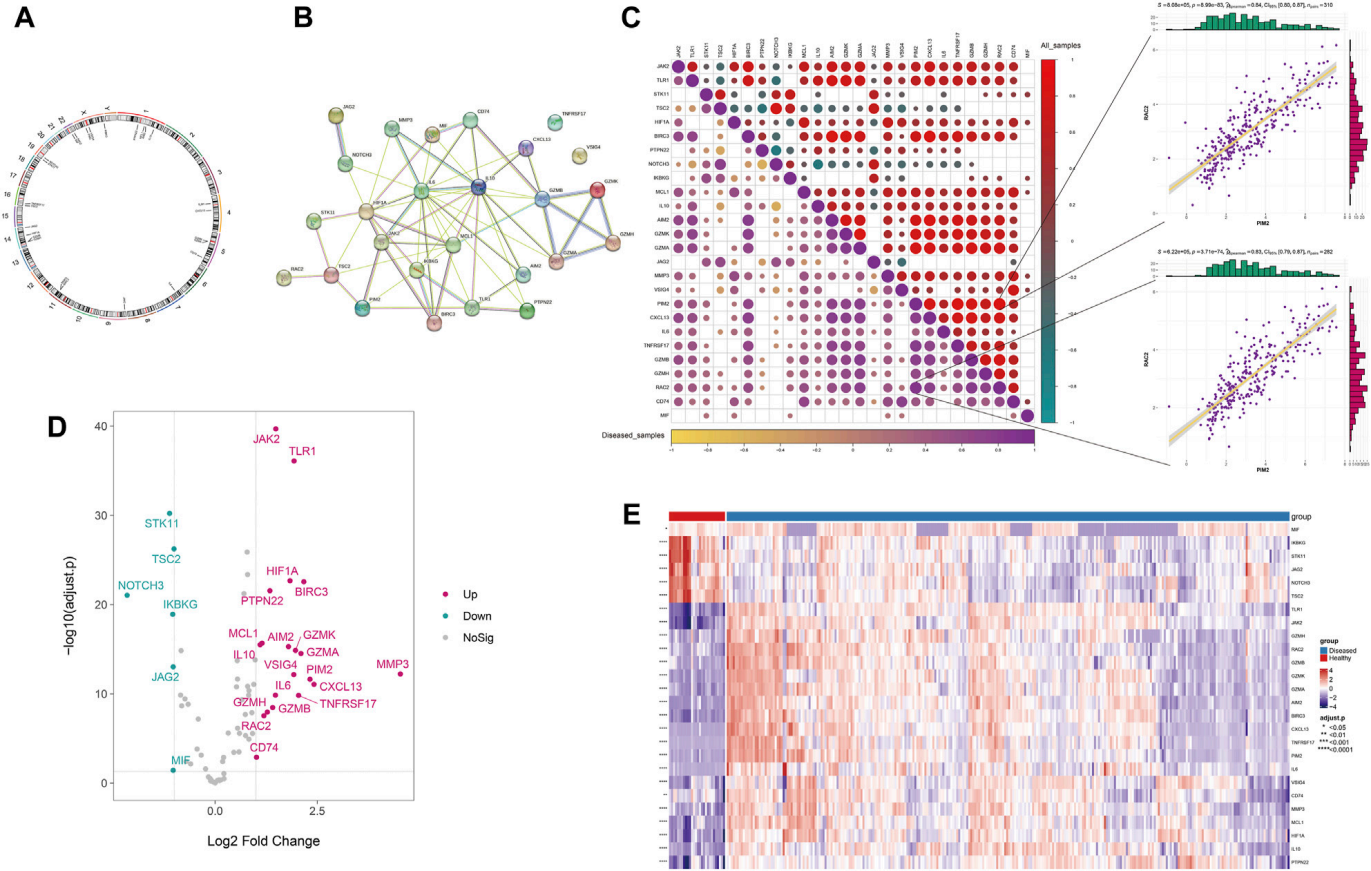
The differential expression of Anoikis-related genes. **(A)** The chromosomal locations of the differentially expressed Anoikis-related genes. **(B)** The protein-protein interaction network among the differentially expressed Anoikis-related genes. **(C)** The Spearman correlation between the differentially expressed Anoikis-related genes in all and disease samples (the lower-left panel shows the correlation heatmap for disease samples, and the upper-right panel shows the correlation heatmap for all samples). **(D)** The volcano plot of differentially expressed Anoikis-related genes. **(E)** The heatmap of differentially expressed Anoikis-related genes.

Based on the integrated expression data, differential analysis was performed between disease and normal samples. The results showed that 26 Anoikis-related genes were differentially expressed between disease and normal samples (*P* < 0.05), with 20 upregulated and 6 downregulated genes (Figure 1D.E), and their chromosomal locations were shown in Figure 1A. The protein interaction network of differentially expressed Anoikis genes revealed that *IL6, MMP3, HIF1A, IKBKG, IL10, MCL1, JAK2*, and others had a higher degree of connectivity (Figure 1B). The results of the expression correlation analysis among differentially expressed genes (disease samples, all samples) showed that the correlations between *PIM2* and *RAC2* were the highest in all samples and disease samples (r = 0.84 and 0.83, *P* = 8.99 × 10^−83^ and 3.71 × 10^*−74*^, respectively) (Figure 1C).

### 3.2 Anoikis-related genes are involved in disease immune regulation

**FIGURE 2:**
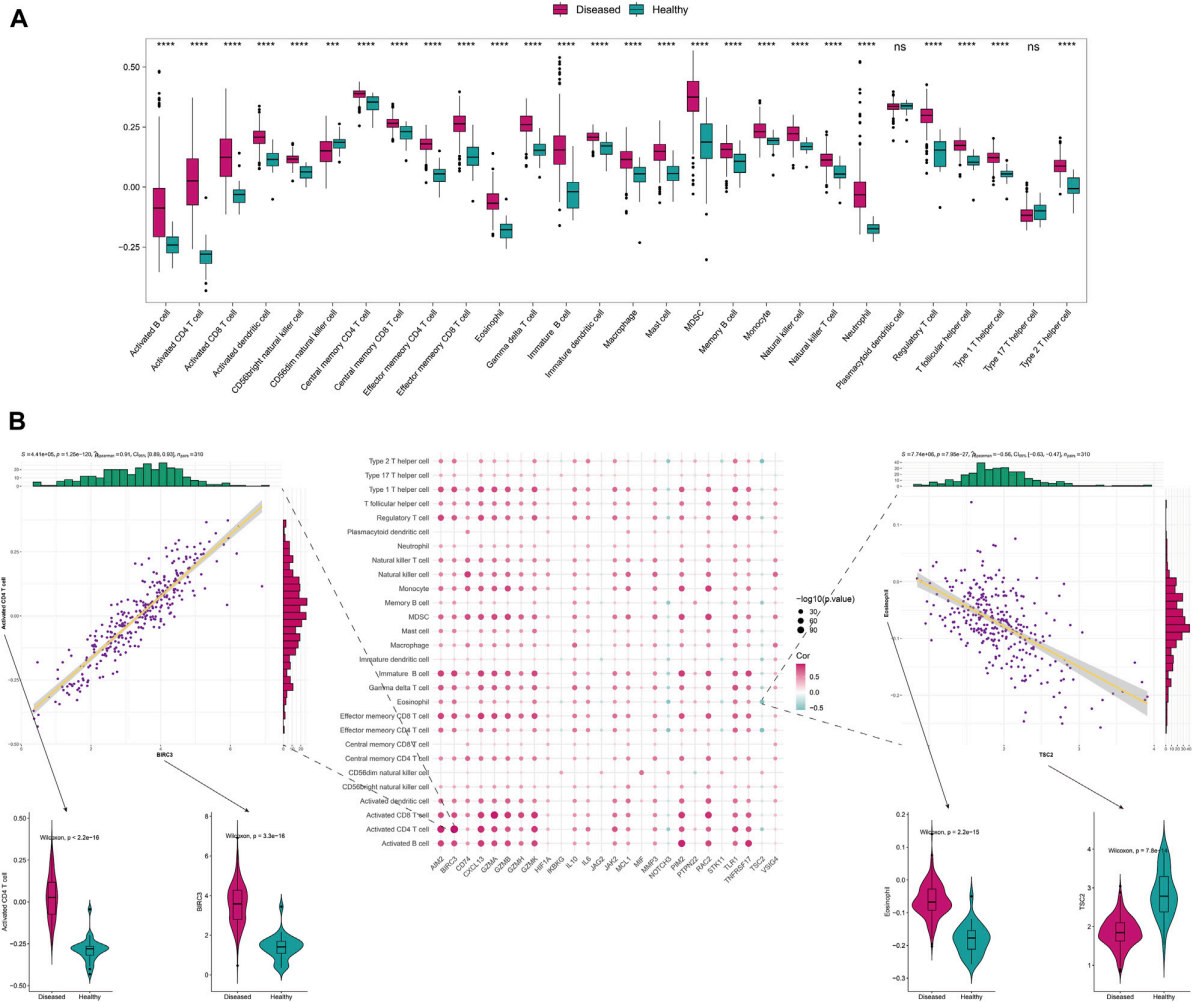
The immune-related analysis of Anoikis-related genes. **(A)** The differences in immune-infiltrating cells between disease and normal samples. **(B)** The Spearman correlation between the differentially expressed Anoikis-related genes and immune cell infiltration scores, displaying the scatter plots of their maximum positive and negative correlations, as well as the boxplots of the immune cells and genes with positive and negative correlations that differ between disease and normal samples.

To further explore the correlation with immune status, we quantified different immune cell subtypes using ssGSEA based on the integrated data and compared the differences in infiltration levels between groups using the rank-sum test. The box plots of the infiltration scores of different immune cells between disease and normal samples showed that most of the immune cells, such as activated B cell, activated CD4+T cell, activated CD8+T cell, and activated dendritic cell, were significantly different between disease and normal groups (*P* < 0.05) (Figure 2A).

We also analyzed the correlation between Anoikis-related differentially expressed genes and immune-infiltrating cells. The two points with the highest positive and negative correlations were selected. Notably, significant positive correlation was observed between *BIRC3* and activated CD4+T cell (r = 0.91, *P* = 1.25 × 10^−120^), while significant negative correlation was observed between TSC2 and Eosinophil (r = −0.56, *P* = 7.95 × 10^−27^) (Figure 2B). Furthermore, we analyzed the correlation between Anoikis-related gene expression and *HLA* gene. Interestingly, most of the *HLA* genes were differentially expressed between the disease and normal groups (*P* < 0.05). Among them, *CD74* and *NOTCH3* were the genes with highest positive and negative correlations with *HLA-DRB1/DMB*, respectively (r = 0.82, *P* = 8.44 × 10^−78^ and r = −0.40, *P* = 4.91 × 10^*−13*^) (Supplementary Figure S2).

### 3.3 The expression of anoikis-related genes stratifies the disease into biologically distinct subtypes

**FIGURE 3:**
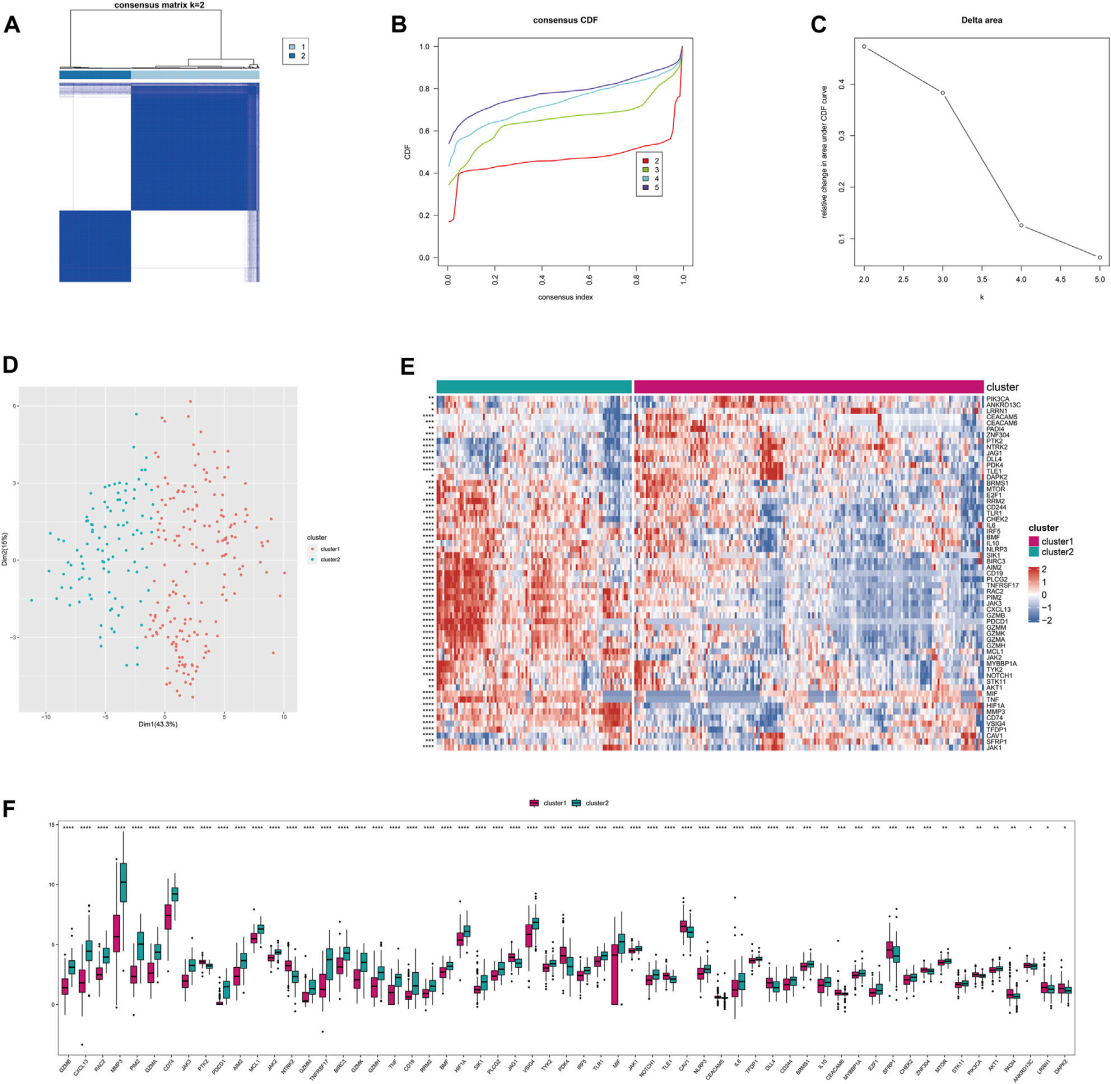
The subtyping of disease samples based on Anoikis-related genes. **(A)** Heatmap of the consistency clustering results matrix. **(B)** Cumulative distribution function (CDF) plot of the consistency clustering results. **(C)** Delta area plot of the consistency clustering results. **(D)** PCA clustering results of the subtyping. **(E)** Heatmap of differentially expressed Anoikis-related genes among the subtypes. **(F)** Boxplot of differentially expressed Anoikis-related genes among the subtypes.

This stratification can be used to reflect similar disease states and help implement personalized treatments. Based on the Anoikis-related differentially expressed genes (26 genes) between RA and normal groups, consistent clustering was performed on the integrated rheumatoid arthritis dataset to identify sample subgroups with similar expression patterns and molecular subtypes based on the optimal clustering K. Here, we identified the 2 subtypes with the most gradual decrease in CDF as the optimal clustering number (Figures 3A–D). Differential analysis was performed on the Anoikis-related genes between subtypes, and 59 differentially expressed Anoikis-related genes (*P* < 0.05) were identified. Heatmaps and boxplots were used to visualize the differentially expressed Anoikis-related genes between subgroups. (Figures 3E,F).

### 3.4 There were functional enrichment differences between different subgroups

**FIGURE 4:**
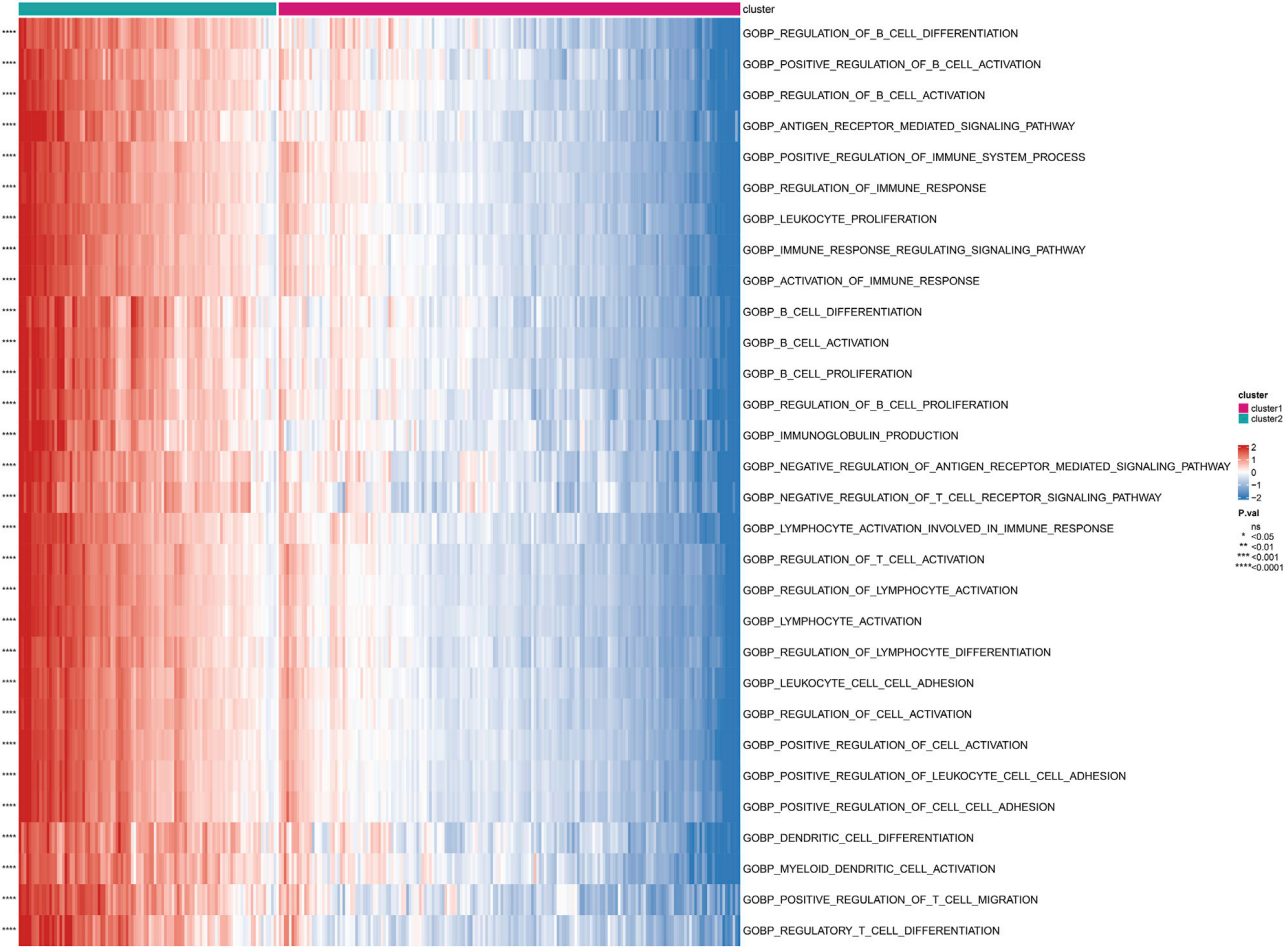
Differential enrichment of GO pathways among subtypes Perform a Gene Ontology (GO) enrichment analysis on differentially expressed genes among subtypes.

**FIGURE 5:**
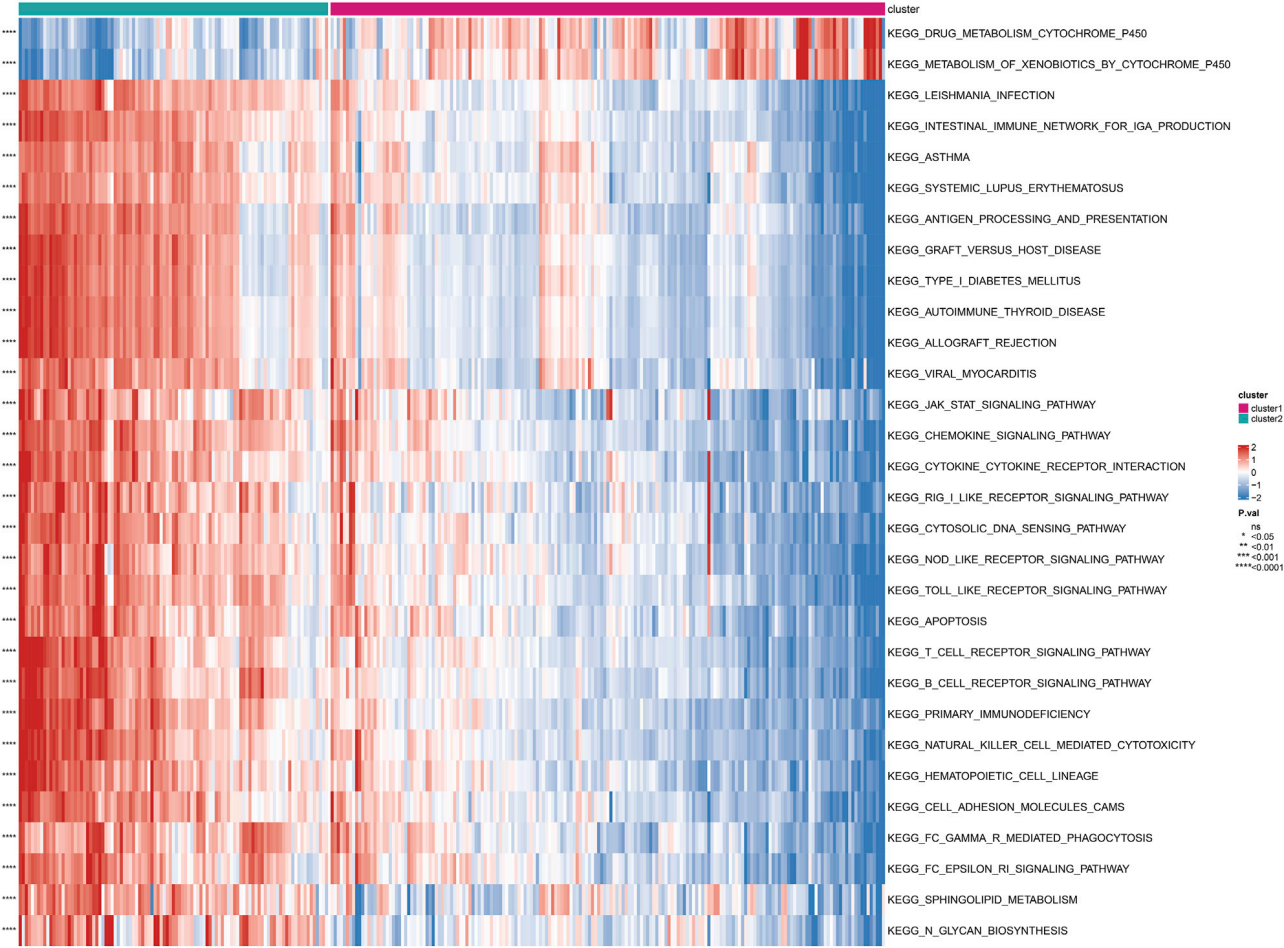
Differential enrichment of KEGG pathways among subtypes Perform a KEGG pathway enrichment analysis on differentially expressed genes among subtypes.

Based on the subgrouping, we calculated the enrichment scores of GO and KEGG pathways between different subgroups, analyzed the enrichment score differences of GO and KEGG pathways between subgroups, and visualized them in heatmaps. Many pathways of GO and KEGG showed significant differences in enrichment scores between subgroups (*P* < 0.05). The top 30 GO processes were mainly related to the proliferation, adhesion, and differentiation reactions of lymphocyte T cells, B cells, and immune cells. The top 30 KEGG pathways also included autoimmune diseases, multiple immune cell receptor pathways, cell apoptosis, and cell adhesion. The enrichment scores of these GO and KEGG pathways in Cluster 2 were significantly higher than those in Cluster 1 (*P* < 0.05) (Figure 4; Figure 5).

### 3.5 The subtypes had different immune characteristics

**FIGURE 6:**
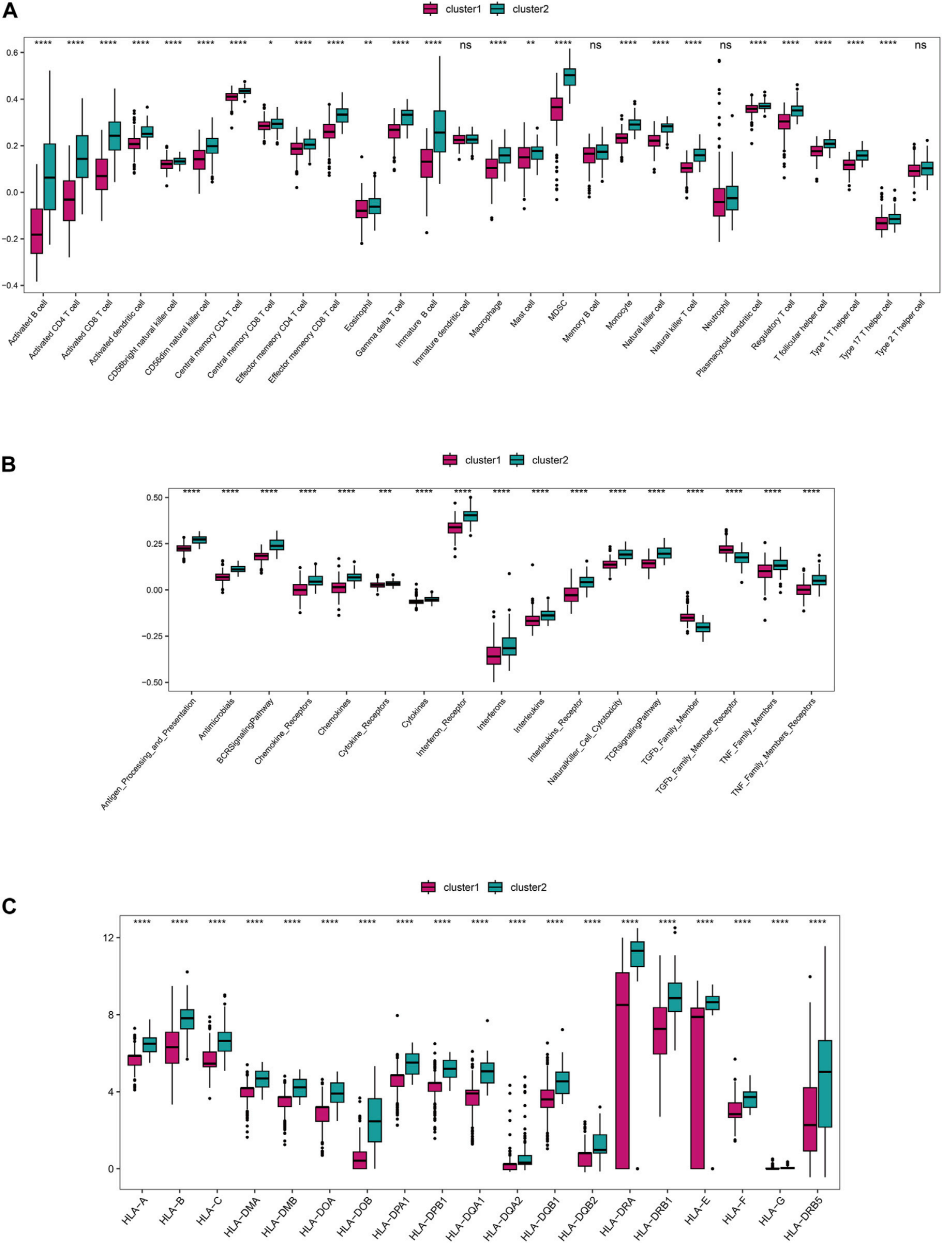
The distinct immune features among subtypes. **(A)** The differences in immune cell infiltration scores (ssGSEA algorithm) among subgroups. **(B)** The differences in enrichment scores (ssGSEA algorithm) of immune response gene sets among subgroups. **(C)** The differential expression of HLA genes among subgroups.

Based on the integrated rheumatoid arthritis data, ssGSEA was used to quantify different immune cell subtypes, and the differences in infiltration levels between subgroups were compared using the rank-sum test. The boxplots of infiltration scores of different immune cells between subgroups showed that most of the immune infiltrations, such as activated B cell, activated CD4+T cell, activated CD8 +T cell, and activated dendritic cell, were significantly different between subgroups (*P* < 0.05), and the immune cell infiltrations in Cluster 2 were significantly higher than those in Cluster 1 (*P* < 0.05) (Figure 6A). We also compared the immune response gene sets between subgroups and found that most immune response gene sets, such as antigen processing and presentation, antimicrobials, and BCR signaling pathway, were significantly different between subgroups, and the immune response gene sets in Cluster 2 were significantly higher than those in Cluster 1 (*P* < 0.05) (Figure 6). In addition, we compared the expression of *HLA* genes between subgroups and found that 19 *HLA* genes were significantly different between subgroups, with the expression of *HLA* genes in Cluster 2 being significantly higher than that in Cluster 1 (*P* < 0.05) (Figure 6).

### 3.6 Identification of key molecules based on co-expression network analysis

**FIGURE 7:**
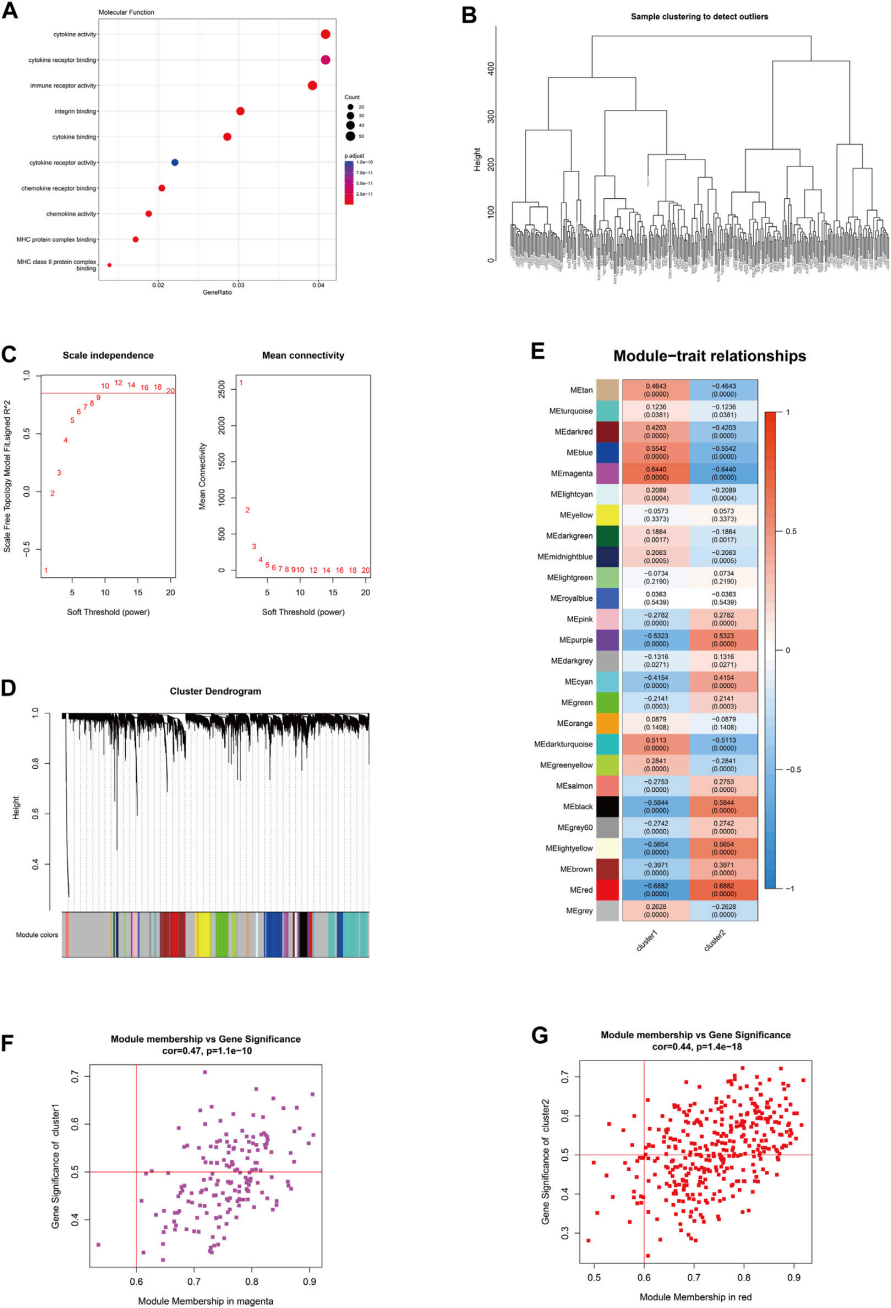
The hub gene selection using WGCNA. **(A)** The molecular functions of differentially expressed genes among subtypes according to GO enrichment analysis. **(B)** The hierarchical clustering tree of WGCNA training samples. **(C)** The different soft thresholds and the corresponding scale-free fitting indices (scale-free R2) where the *x*-axis represents the different soft thresholds and the *y*-axis represents the corresponding scale-free fitting indices. **(D)** Gene hierarchical clustering dendrogram and modules constructed by WGCNA, where the gray nodes in the color bar indicate genes not assigned to any module, and the remaining colors represent the built modules. **(E)** Heatmap showing the module-trait correlation. **(F)** The magenta module with the highest positive correlation in cluster 1. **(G)** The red module with the highest positive correlation in cluster 2.

Based on the subgrouping, the R package limma was used to calculate the differentially expressed genes between subgroups, and 1,295 differentially expressed genes were identified (*FDR* <0.05 and |log2FC|>0.585). Based on the merged data of rheumatoid arthritis, the R package WGCNA was used to construct a weight co-expression network. First, the data was filtered with the method set to “ward.D2”. Studies have shown that the co-expression network complies with the scale-free network, where the logarithm of the number of nodes with a connectivity of k (log(k)) is negatively correlated with the logarithm of the probability of the node appearing (log (P(k))) and the correlation coefficient is greater than 0.85. To ensure that the network is a scale-free network, the optimal β = 10 was selected (Figure 7). Next, the expression matrix was transformed into an adjacency matrix and then into a topological matrix. Based on TOM, the average-linkage hierarchical clustering method was used to cluster genes and the standard of mixed dynamic tree cut was set, with each gene module having a minimum number of 30 genes. After determining the gene modules using the dynamic tree cut method, the eigengenes for each module were calculated and the modules were subjected to cluster analysis with a height set to 0.25. Modules that were close in distance were merged into new modules. The Pearson correlation coefficient between the ME of each module and the sample phenotype features was calculated, with a higher value indicating greater importance. In Figure 7E, rows represent the eigengenes of each module, columns represent the sample phenotype features, with red indicating positive correlation and blue indicating negative correlation. The higher the color intensity, the higher the correlation. Based on cluster1, the magenta module with the highest positive correlation was selected, and based on cluster2, the red module with the highest positive correlation was selected. Using module membership (MM) > 0.6 and gene significance (GS) > 0.5, 72 and 200 core genes were respectively screened from the two modules (Figures 7F–G). These genes were intersected with the differentially expressed genes between the subtypes mentioned above, resulting in 51 hub cluster1 differential genes and 172 hub cluster2 differential genes (Figure 7).

To further elucidate biological functions of hub genes within distinct clusters, we use GO and KEGG enrichment analyses. The enrichment results revealed that cluster 1 was primarily associated with cellular components such as the basal cortex, collagen-containing extracellular matrix, cell leading edge, and basal part of the cell (*FDR* < 0.05). Additionally, it showed significant involvement in signaling pathways like the Hippo signaling pathway across multiple species (*FDR* < 0.05). Cluster 2 exhibited significant enrichment in biological processes, including lymphocyte differentiation, mononuclear cell differentiation, and T cell differentiation (*FDR* < 0.05). In terms of cellular components, it was associated with the immunological synapse, plasma membrane signaling receptor complex, and phagocytic vesicle (*FDR* < 0.05). Moreover, molecular functions related to cytokine receptor activity, cytokine binding, and immune receptor activity were enriched (*FDR* < 0.05). Cluster 2 also demonstrated significant involvement in signaling pathways such as Th17 cell differentiation, Natural killer cell-mediated cytotoxicity, and the TNF signaling pathway (*FDR* < 0.05) (Supplementary Figure S3). We further validated 42 differential genes from 51 hub cluster 1 and 153 differential genes from 172 hub cluster 2, showing significant differential expression (*FDR* <0.05) in synovial tissues of both RA and OA (Table 2) (Supplementary Table S1).

**TABLE 2:** The information on hub key genes and their interacting drugs.

	Cluster1	Cluster2
Number of hub genes	72	200
Number of hub differential genes	51	172
Key drug interaction genes	6	6
Number of genes detected by DGIDB	3	5
Number of drug interactions	187	57

### 3.7 Potential treatment strategies

**FIGURE 8:**
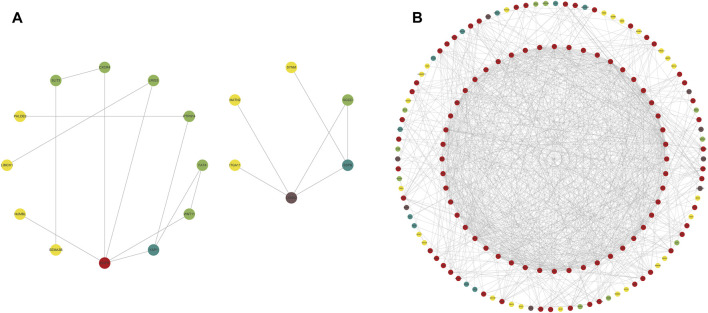
Hub differential gene nodes in the PPI network. **(A)** Hub differential genes in cluster 1. **(B)** Hub differential genes in cluster 2. (The colors gradually change from red to yellow, indicating the changes in degree from large to small, where red is the largest, and yellow is the smallest.).

**FIGURE 9:**
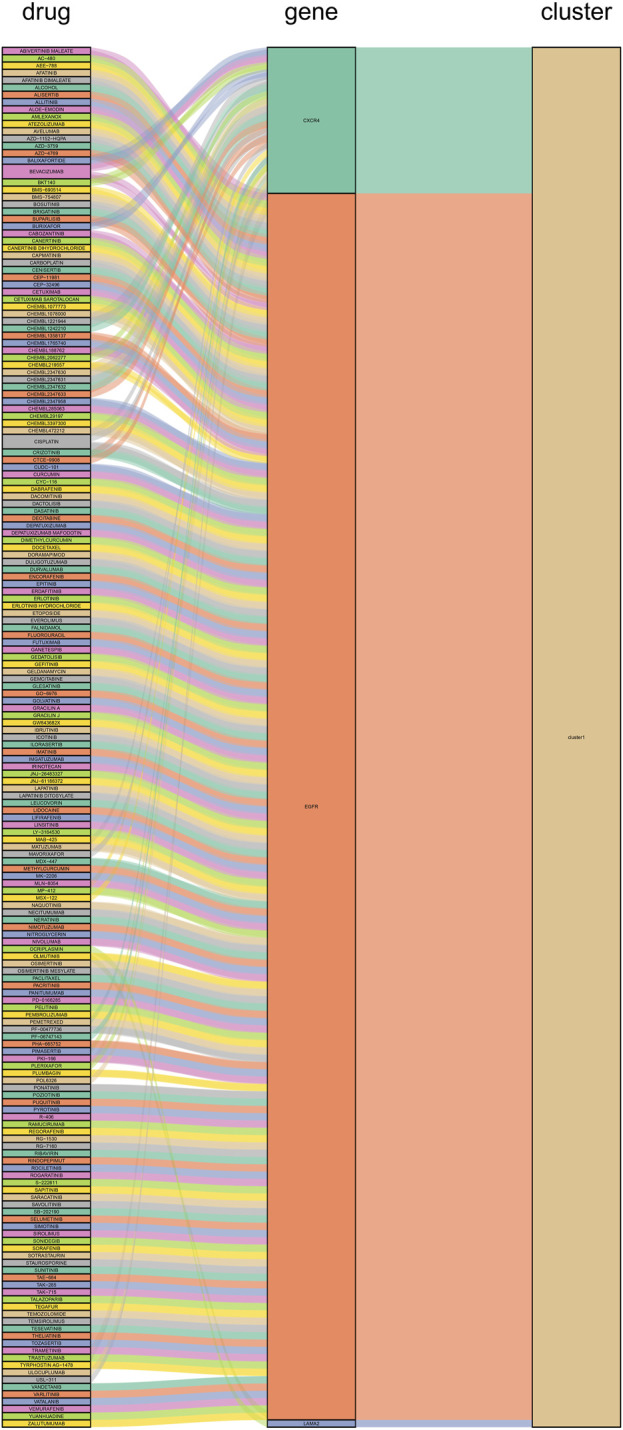
Interactions between cluster 1 hub differential genes and drugs. Utilize hub genes identified by Cluster 1 and perform an analysis of the interaction between the hub genes and drugs based on a drug database, followed by displaying the results.

**FIGURE 10:**
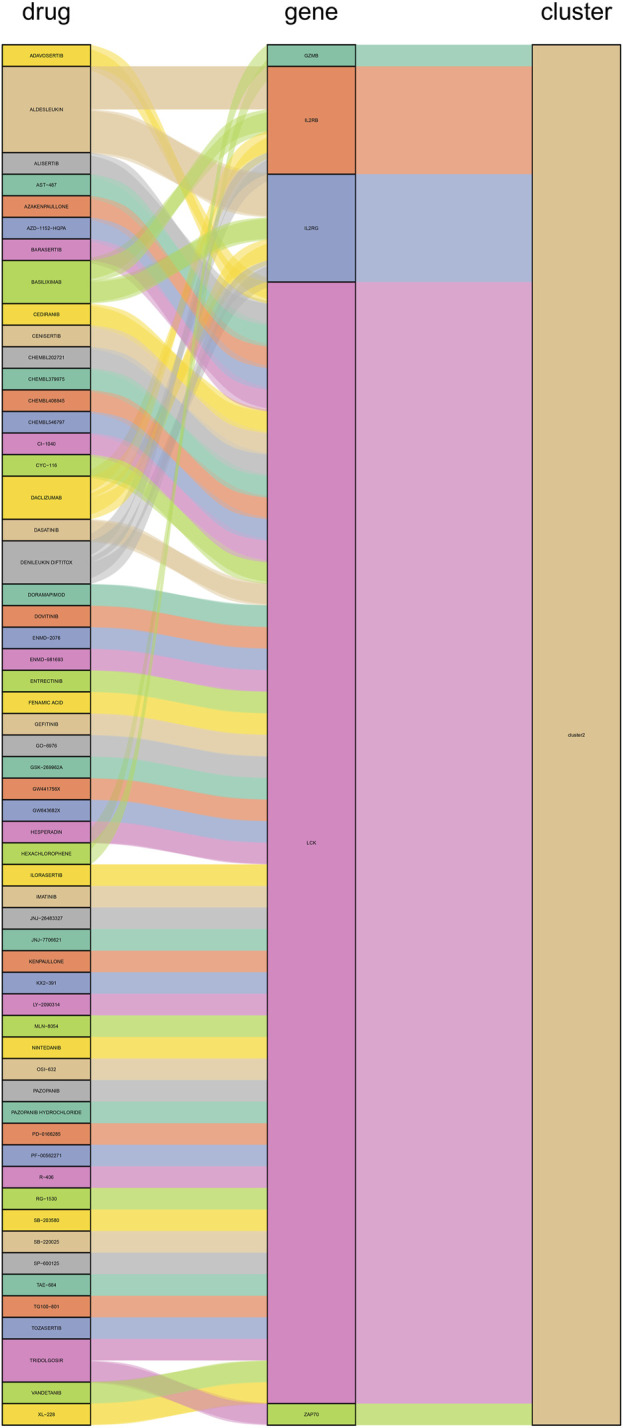
Interactions between cluster 2 hub differential genes and drugs. Utilize hub genes identified by Cluster 2 and perform an analysis of the interaction between the hub genes and drugs based on a drug database, followed by displaying the results.

We constructed PPI networks for the 51 cluster1 hub differential genes and the 172 cluster2 hub differential genes, respectively (Figure 8). Cytoscape was used to identify hub nodes (key genes selected by degree for each cluster) in the PPI networks, which were then used for gene-drug interactions. The top 6 genes with the highest degree (top 4 genes with degree of 2–5) were selected as key genes for gene-drug interactions in cluster1. Next, we mined gene-drug interaction relationships based on the DGIdb database v4.2.0 (https://www.dgidb.org/), displaying unique gene-drug interactions with 3 genes and 187 drugs, and a Sankey diagram was drawn to illustrate the interactions (Figure 9). Similarly, the top 6 genes with the highest degree (top 6 genes with degree of 43–63) were selected as key genes for gene-drug interactions in cluster2. Gene-drug interaction relationships were mined based on the DGIdb database v4.2.0 (https://www.dgidb.org/), displaying unique gene-drug interactions with 5 genes and 57 drugs, and a Sankey diagram was drawn to illustrate the interactions (Figure 10).

## 4 Discussion

Rheumatoid arthritis (RA) is a self-immune disease that seriously endangers the physical and mental health of patients, and can involve multiple systems in the body and have various complications. The existing first-line therapies are not sufficient to achieve clinical cure for all patients, and some patients may experience adverse reactions due to the complexity of the pathogenesis. Therefore, a thorough understanding and research on the pathogenic mechanisms of RA is of great significance for the development of clinical strategies for RA. Anoikis, as an important mechanism of cell death, may play a role in RA through mechanisms that promote cell death, particularly in cases where fibroblast-like synoviocytes (FLS) exhibit resistance to anoikis, which may lead to RA disease progression.

Firstly, we analyzed the differential expression of anoikis-associated genes between disease and normal samples and conducted protein interaction analysis, revealing that IL6, MMP3, HIF1A, IKBKG, IL10, MCL1, JAK2, and other genes may interact with each other. The *IL-6/JAK2/STAT3/VEGF* pathway is also a key pathway for promoting FLS proliferation and angiogenesis in RA (Cheng et al., 2020), where IL-6 can promote MMP3 secretion leading to bone destruction (Takeuchi et al., 2021). Several biologics have been used for the treatment of IL6-related autoimmune diseases, such as Tocilizumab, Siltuximab, and Sarilumab. These medications are biologic agents that target the IL6 signaling pathway and have been clinically used for treating various autoimmune diseases associated with IL6, including rheumatoid arthritis, systemic sclerosis, giant cell arteritis, and juvenile idiopathic arthritis. They work by blocking the IL6 signaling pathway through targeting the IL-6 receptor or binding directly to IL-6, thus inhibiting inflammation and immune reaction to alleviate disease symptoms and control disease progression. HIF1A has also been reported to be associated with angiogenesis and inflammation in RA (Brouwer et al., 2009), where TNF can induce glucose metabolism transition of FLS through *GLUT1* and *HIF1A* (Koedderitzsch et al., 2021). *IKBKG* and *MCL1* belong to the crucial part of pro-survival pathway proteins, which may contribute to the resistance of FLS to anoikis-apoptosis and promote proliferation (Jiao et al., 2018). Additionally, we found that *RAC2* and *PIM2* had the highest correlation in disease samples and all samples. *RAC2* is significantly upregulated in the inducible nitric oxide synthase (iNOS) regulated NO production process in RA synovium (Dey et al., 2016); whereas, our previous unpublished results showed that overexpression of *PIM2* may promote inflammation by promoting synovial proliferation. The high correlation between *RAC2* and *PIM2* may be reflected in the synergistic regulation of FLS proliferation. The differential expression of these genes may represent a pattern of regulating FLS proliferation, inflammation, and bone destruction.

In order to further explore the correlation between these genes and immunity, we conducted immune infiltration analysis, and found that most immune cells, including activated B cell, activated CD4+T cell, activated CD8+T cell, and activated dendritic cell, showed significant differences between disease and normal groups. As a type of autoimmune disease, RA involves various immune cells infiltrating in the local joint microenvironment, which together promote the progression of RA (Zhao et al., 2021; Zhao et al., 2022c). We further analyzed the correlation between Anoikis-related differentially expressed genes and immune infiltration cells, among which the correlation between *BIRC3* and *TSC2* with activated CD4+T cell and Eosinophil was the highest positive or negative correlation relationship. BIRC3 is a survival-promoting protein and a downstream target of ATF6a. It significantly increases in the RA synovium and CIA animal model, and its positive expression correlation with activated CD4^+^ T cells may imply that BIRC3 positively promotes the survival of autoimmune T cells (Ge et al., 2022). Although Eosinophil is considered to induce inflammation in asthma, it may have a suppressive effect on inflammation in RA by increasing the differentiation of anti-inflammatory macrophages (Chen et al., 2016; Andreev et al., 2021). The relationship between TSC2 and Eosinophil and RA has not been studied in detail. TSC2 mainly participates in the mTOR-mediated cellular autophagy pathway, and further studies are needed to investigate the relationship among them (Miao et al., 2019).

The estimated heritability of RA patients is 40%–60%, of which 10%–40% is contributed by *HLA* genes. HLA genes exhibits high variability, both in terms of the classical locus of *HLA* class I and class II genes. These genes translate proteins that process antigen peptides and present them to other cells of the immune system. *HLA* class I genes mainly include *HLA-A, HLA-B*, and *HLA-C,* while *HLA* class II genes mainly include *HLA-DRB1*, *HLA-DQB1*, and *HLA-DPB1* (Ali and Vino, 2016). Therefore, we further analyzed the differential expression of *HLA* genes and their correlation with Anoikis-related genes. We found that most *HLA* genes showed differential expression between the normal and disease groups, mainly including*HLA-A/B/DMA/DMB/DOA/DPA1/DPB1/DQA1/DQA2/DQB1/DQB2/DRA/DRB1/F/DRB5*. In addition, *CD74* and *NOTCH3* exhibited the highest positive or negative correlation with *HLA-DRB1/DMB*, respectively. CD74 is a membrane protein mainly distributed on the surface of immune cells, which transduces signals by binding to MIF (Sánchez-Zuno et al., 2021), while *HLA-DRB1* sharesepitope sequences that mainly encode antigen presentation proteins and contribute to ACPA-positive RA (Kampstra and Toes, 2017). The positive correlation between the two may reflect the degree of immune cell-mediated autoimmune inflammation. *NOTCH3* and NOTCH target genes are significantly upregulated in FLS, and in animal models, inhibition of *NOTCH3* or blocking NOTCH signaling can reduce inflammation and prevent joint damage in inflammatory arthritis (Wei et al., 2020). *HLA-DMA*0103 and HLA-DMB0104 alleles are considered to be biomarkers reflecting the severity of RA disease (Morel et al., 2004), but further experimental evidence is needed to confirm the negative correlation between *NOTCH3* and *HLA-DMB*.

The use of differential genes for disease typing is beneficial for individualized treatment. Based on the 26 differential genes associated with Anoikis, we further divided the disease group into two clusters (cluster 1 and cluster 2). Functional enrichment, immune infiltration, and *HLA* genotyping analyses were then conducted. We found that most immune cells showed significantly differential expression between the two clusters, with the majority of immune cells exhibiting significantly higher scores in cluster 2 than in cluster 1. Consistently, most immune response gene sets, such as antigen processing and presentation, antimicrobials, and BCR signaling pathway, as well as HLA gene expression, were significantly higher in cluster 2 than in cluster 1, with statistical differences. In summary, this may indicate that cluster 2 has a more severe inflammatory response and disease severity.

Subsequently, we conducted WGCNA analysis, which is a system biology method that uses gene expression data to construct an unscaled network. We first found a large number of differential genes between the two clusters, and GO enrichment analysis showed that the major pathological factors of RA were mainly related to cytokines and cytokine receptors. Through WGCNA analysis and intersecting with the aforementioned differential genes, we obtained 51 hub cluster 1 differential genes and 172 hub cluster 2 differential genes. Based on these cluster-specific differentially expressed genes, we initially conducted GO and KEGG enrichment analyses to further elucidate their biological functions. Cluster 1 was notably associated with the Hippo signaling pathway, a crucial factor influencing RA FLS behavior. Within this pathway, the tyrosine phosphatase PTPN14 has been reported to enhance the pathological manifestations of RA FLS by forming a complex with YAP (Bottini et al., 2019). Cluster 2’s enrichment results revealed associations with a multitude of lymphocytes and related signaling pathways. Lymphocytes, including T cells, are pivotal effector cells in RA inflammation and its subsequent progression. Subsequently, we conducted additional validation of the identified cluster genes in synovial tissue from both RA and OA patients. This validation process included 42 hub genes from cluster 1 and 153 hub genes from cluster 2. These genes were identified as significant differential hub genes in OA or HC and RA respectively, marking them as pivotal genes distinguishing the two clusters. These genes represent important directions for our future research, warranting further elucidation of their functionality and underlying mechanisms. We further analyzed protein-protein interactions and mined core hub genes. We also explored the interactions between these hub genes and drugs based on the *DGIdb* database. We identified three hub genes that interact with drugs in cluster 1, namely, *CXCR4, EGFR*, and *LAMA2*, and five hub genes that interact with drugs in cluster 2, namely, *GZMB, IL2RB, IL2RG, LCK*, and *ZAP70*. CXCR4 is aberrantly expressed in multiple immune cell populations in RA and has multiple functions, including promoting FLS proliferation, facilitating T cell migration, promoting the differentiation of inflammatory macrophages, and promoting inflammation, angiogenesis, and bone destruction processes (Zhao et al., 2022a). EGFR has long been considered a potential therapeutic target for RA, as its activation primarily promotes synoviocyte proliferation and cytokine production, and EGFR activation is also an important mechanism by which cells resist apoptosis, which may contribute to FLS overproliferation (Yuan et al., 2013). LAMA2 is primarily associated with congenital muscular dystrophies (CMD) (Barraza-Flores et al., 2020). LAMA2 is a major component of the basement membrane and an extracellular protein that mediates cellular adhesion, migration, and other functions through its interactions with other extracellular matrix molecules. As there is a lack of direct research on the relationship between LAMA2 and RA, further experimental studies are needed to establish a link between the two. Given that LAMA2’s physiological functions, it may affect the proliferation and migration of multiple cells, including FLS, via apoptosis resistance. GZMB is mainly associated with inflammation and matrix degradation and is the strongest apoptotic activity of the granzyme family members, with caspase-like abilities. In RA, GZMB is abnormally expressed in multiple immune and non-immune cells, which may contribute to bone destruction through mechanisms such as matrix degradation (Zheng et al., 2023a). Studies have shown that GZMB can increase tissue-destructive effects by inducing apoptosis, contributing to the pathological characteristics of RA (Buzza et al., 2005). IL2RB is a subunit of the IL-2 receptor that mainly participates in T cell-mediated immune responses and is associated with early bone erosion in RA as a susceptibility gene (Ruyssen-Witrand et al., 2014). The protein encoded by IL2RG is an important signaling component of many interleukin receptors, including interleukin-2, -4, -7, and -21, and may participate in multiple signaling pathways in RA. *LCK* and *ZAP70* mainly participate in the development and activation of lymphocyte T and B cells, promoting the abnormal immune state in RA. *LCK* can bind to cell surface receptors (including CD4 and CD8) and other signaling molecules (Hu et al., 2022), while mutations in *ZAP70* change the sensitivity of developing T cells to thymic positive/negative selection by altering self-peptide/MHC complexes, change the self-reactive TCR repertoire to include dominant arthritis-specific ones, and affect thymic development and the production of self-immune inhibitory regulatory T cells (Treg) (Takeuchi et al., 2020).

Finally, we discussed the relevance between key hub genes in the two clusters and potential drug efficacy. In cluster 1, the major drug-related genes included *CXCR4, EGFR*, and *LAMA2*. Targeting the inhibition of CXCR4 and EGFR may be crucial directions to suppress the aberrant pathological behavior of RA FLS and angiogenesis. For example, BEVACIZUMAB can counteract angiogenesis by inhibiting VEGF (Mitsuhashi et al., 2015), while CXCR4 antagonists exhibit stronger anti-angiogenic effects (Gravina et al., 2017). OLMUTINIB is also an EGFR inhibitor (Roskoski, 2019), and given the biological functions associated with LAMA2, it suggests that targeting LAMA2 and EGFR may hold therapeutic value for RA. In cluster 2, the major drug-related genes included *GZMB, IL2RB, IL2RG, LCK*, and *ZAP70*. The connection between the GZMB family and RA has been progressively elucidated (Zheng et al., 2023b). DACLIZUMAB is a specific IL2 receptor-targeting drug, which is related to GZMB, IL2RB, and IL2RG, and research indicates its therapeutic potential in the experimental arthritis model induced by CIA (Brok et al., 2001). It is possible that innovative treatments for RA targeting IL2 may emerge in the future. As for LCK and ZAP70, as previously mentioned, they are primarily associated with lymphocytes, and numerous drugs target these genes, potentially impacting various lymphocytes. This naturally affects the abnormal inflammation and bone destruction in RA, although further exploration is needed to determine if targeting LCK and ZAP70 for drug development is a viable approach.

In summary, our analysis revealed that the hub genes in cluster 1 mainly function in the excessive proliferation of FLS in early RA and the autoimmune cell migration process, while the hub genes in cluster 2 mainly function in the excessive activation of autoimmune cells and bone destruction processes in later stages of RA. Compared to cluster 1, cluster 2 represents a more severe RA state. Moreover, these key hub genes in the clusters are also associated with some clinical drugs, including common clinical drugs for RA, which can provide guidance for the clinical treatment and target development of RA.

## Data Availability

The original contributions presented in the study are included in the article/Supplementary Material, further inquiries can be directed to the corresponding author.

## Supplementary material

SUPPLEMENTARY FIGURE S1 Flowchart of analysis process. Download relevant datasets from GEO, and perform batch effect removal analysis to obtain corresponding RA and HC samples, then conduct subsequent analysis on the merged sample data.

SUPPLEMENTARY FIGURE S2 The differential analysis of HLA genes in Anoikis-related genes. A: The differences in HLA gene expression between disease and normal samples. B: The Spearman correlation between differentially expressed Anoikis-related genes and HLA gene expression, displaying the scatter plots of their maximum positive and negative correlations and the boxplots of the positive and negative correlations of Anoikis-related genes and HLA genes that differ between disease and normal samples.

SUPPLEMENTARY FIGURE S3 GO and KEGG for hub genes in cluster 1 and cluster 2 A: GO results for hub genes in cluster 1. B: KEGG results for hub genes in cluster 1.C: GO results for hub genes in cluster 2. D: KEGG results for hub genes in cluster 2.

SUPPLEMENTARY TABLE S1 Validations for hub genes in cluster 1 and cluster 2. The expression of hub genes in cluster 1 and cluster 2 was analyzed in clinical synovial tissue samples collected from the Guanghua Hospital Precision Medicine Research Cohort, which includes 9 rheumatoid arthritis (RA) patients and 15 osteoarthritis (OA) patients, and significant differences were observed (FDR <0.05).
