# Supplementary figures and images for: Identification of immunological characterization and Anoikis-related molecular clusters in rheumatoid arthritis
Source: Front Mol Biosci. 2023 Nov 17;10:1202371. doi: 10.3389/fmolb.2023.1202371 (PMC10691379; doi:10.3389/fmolb.2023.1202371)

A

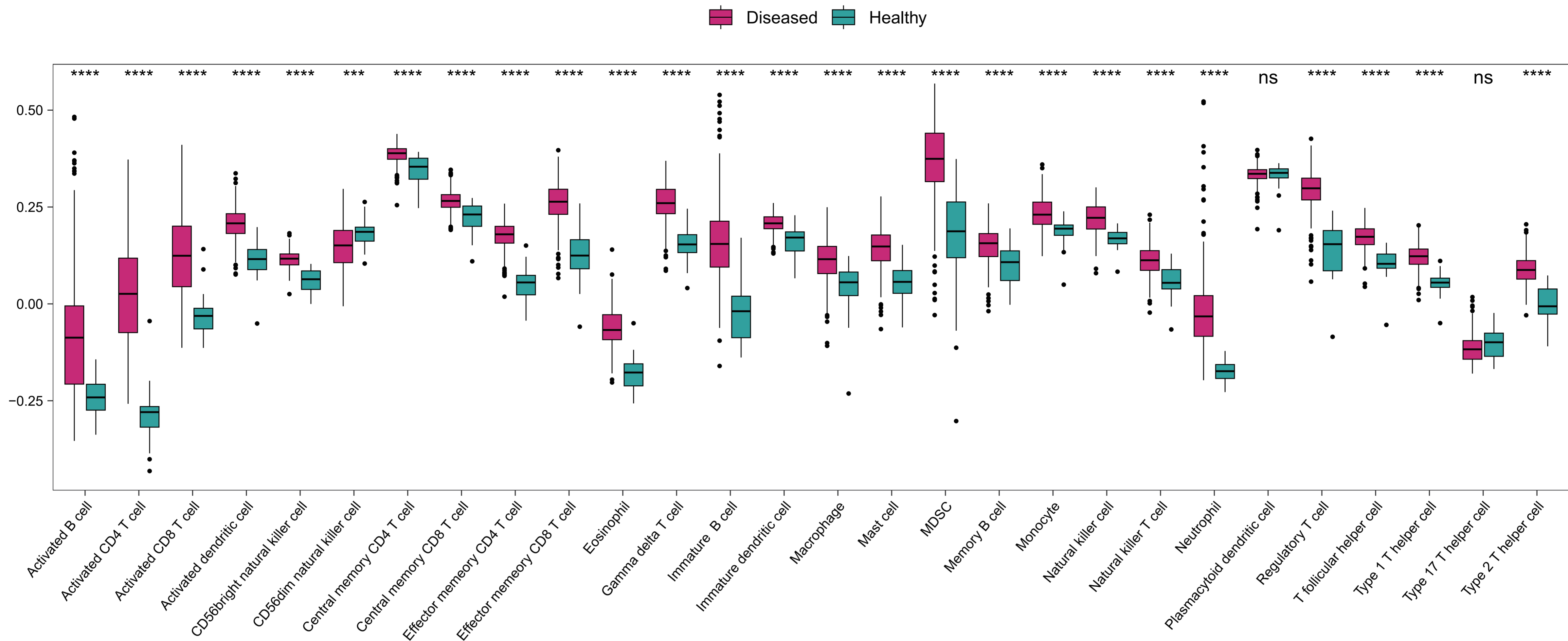

B

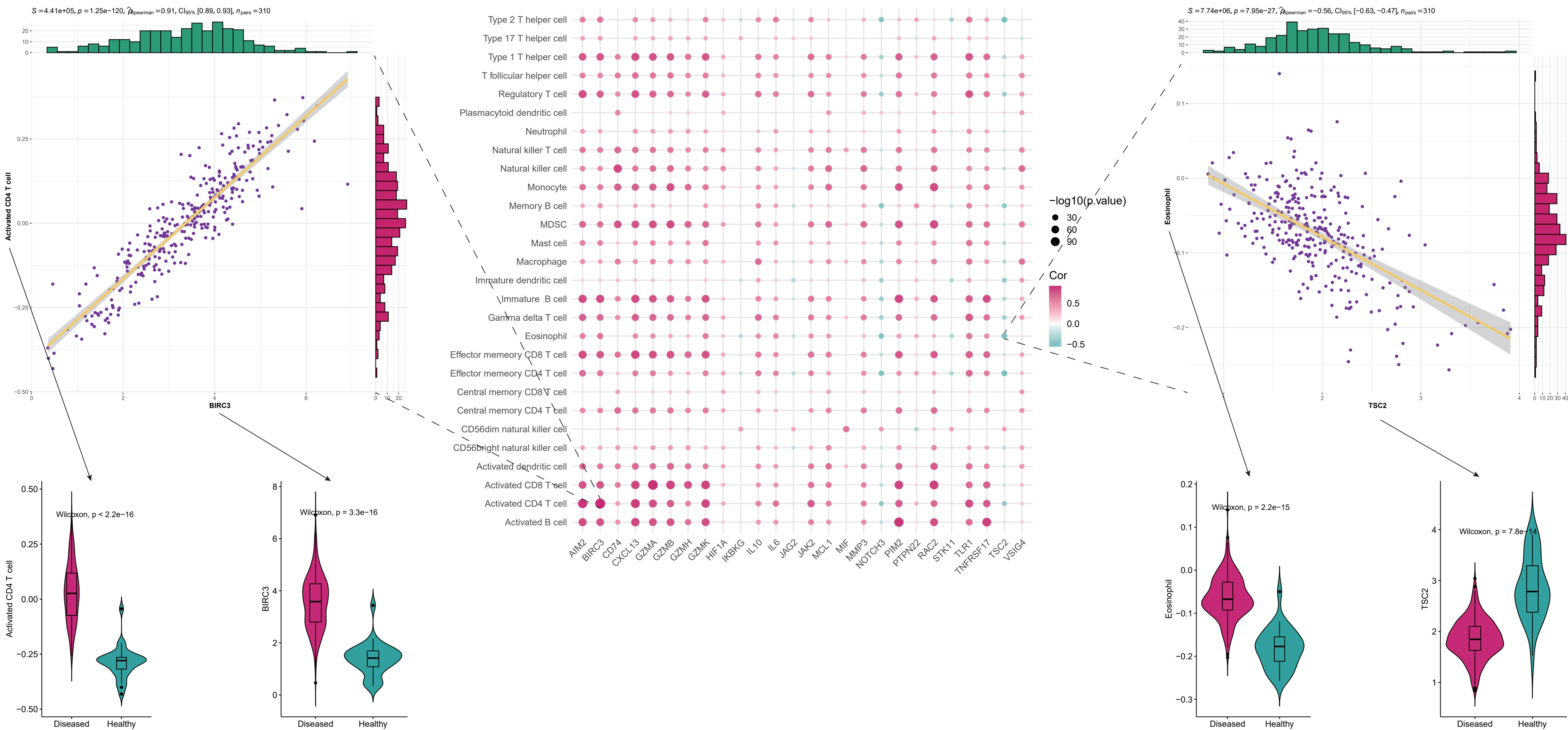

Supplement: Supplementary file 1 [file DataSheet7.PDF]

A

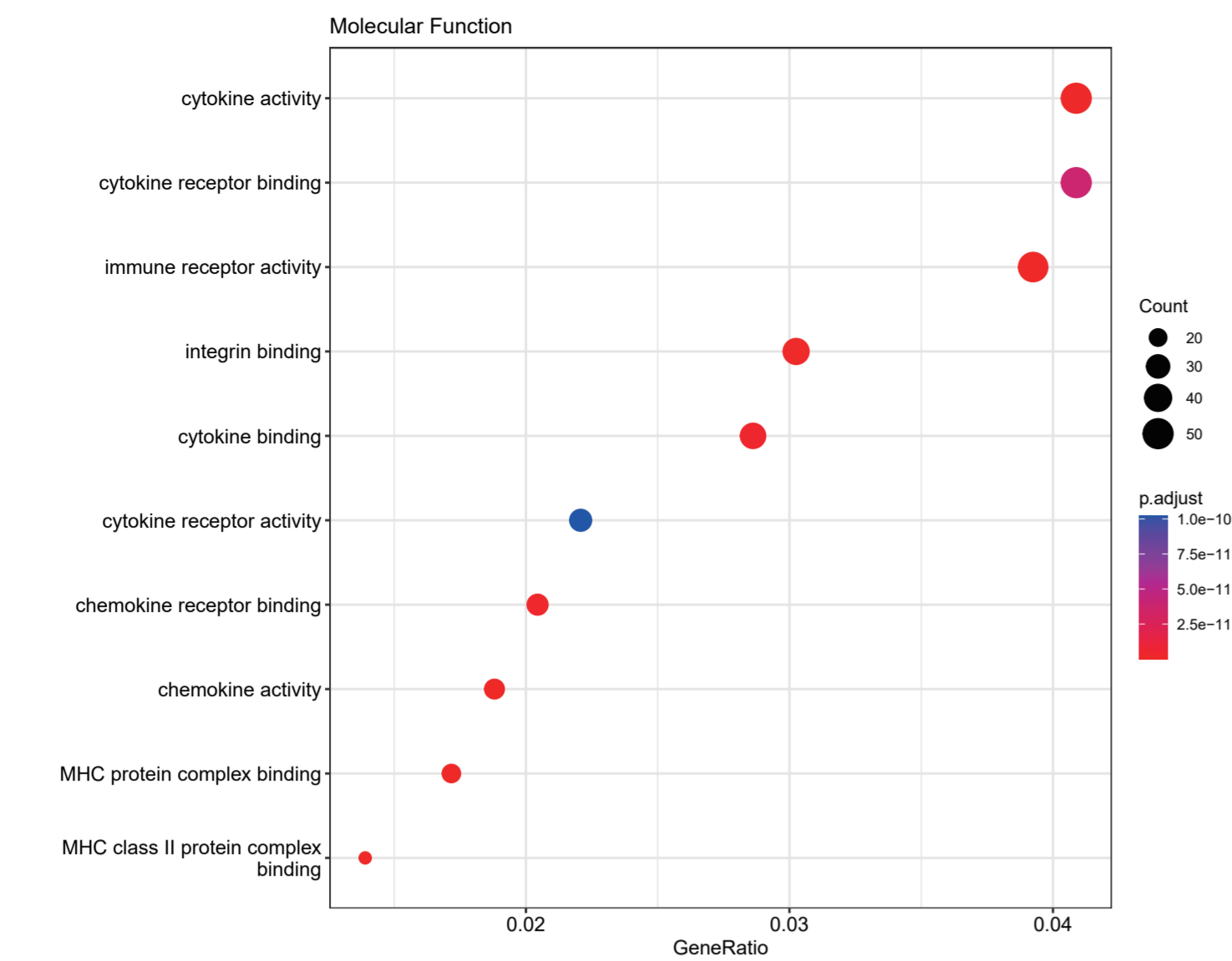

B

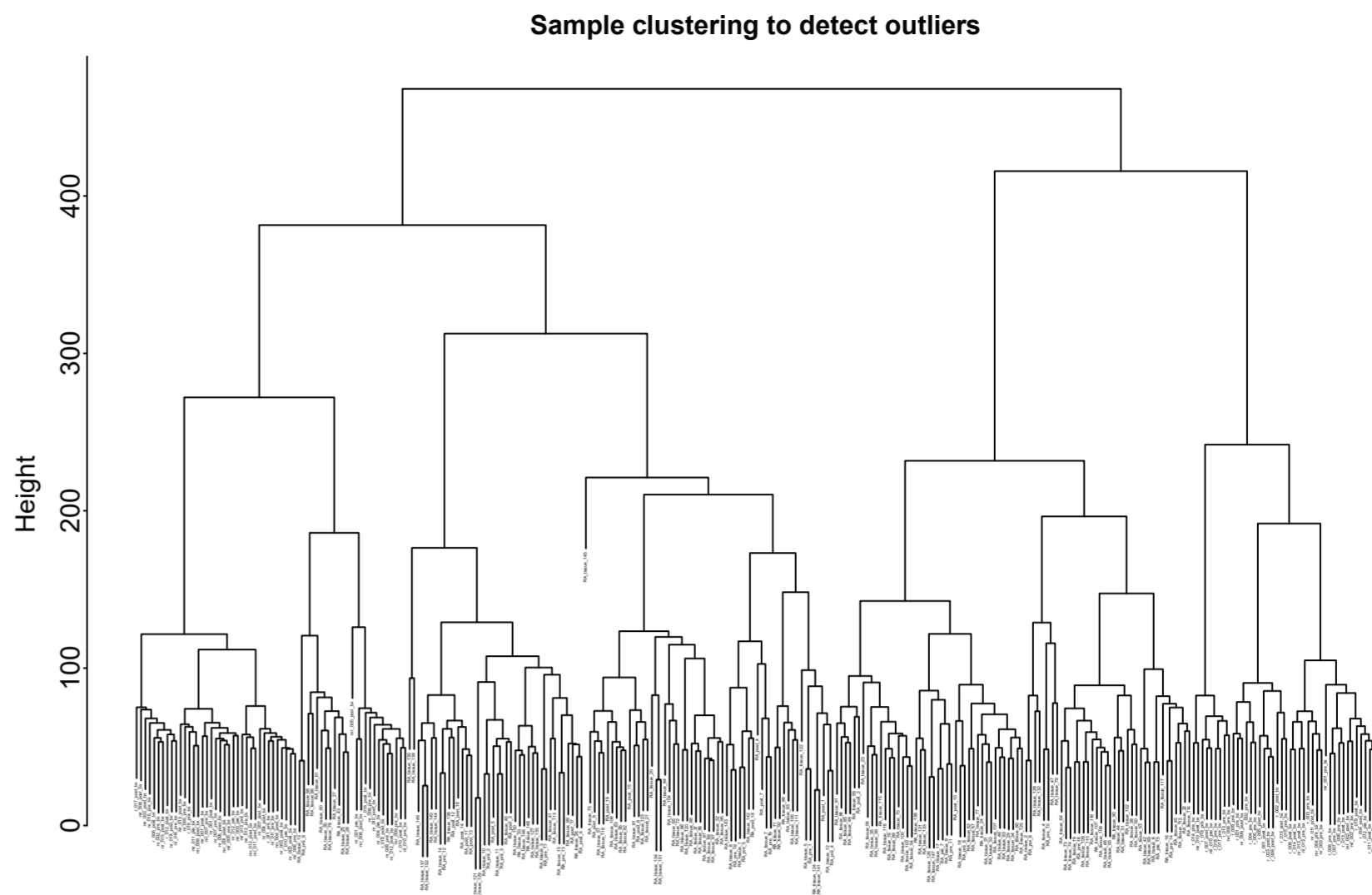

C

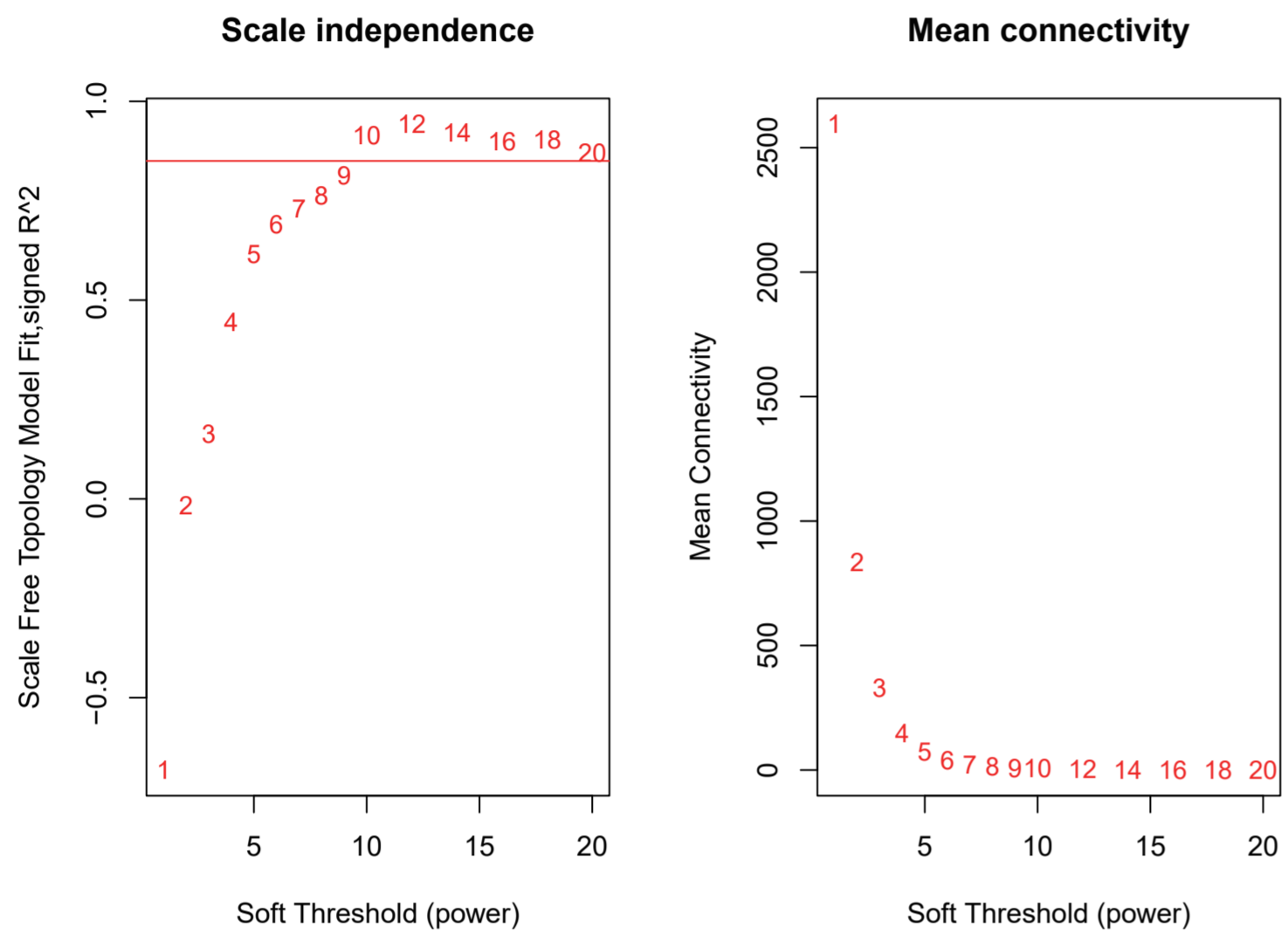

E

### Module-trait relationships

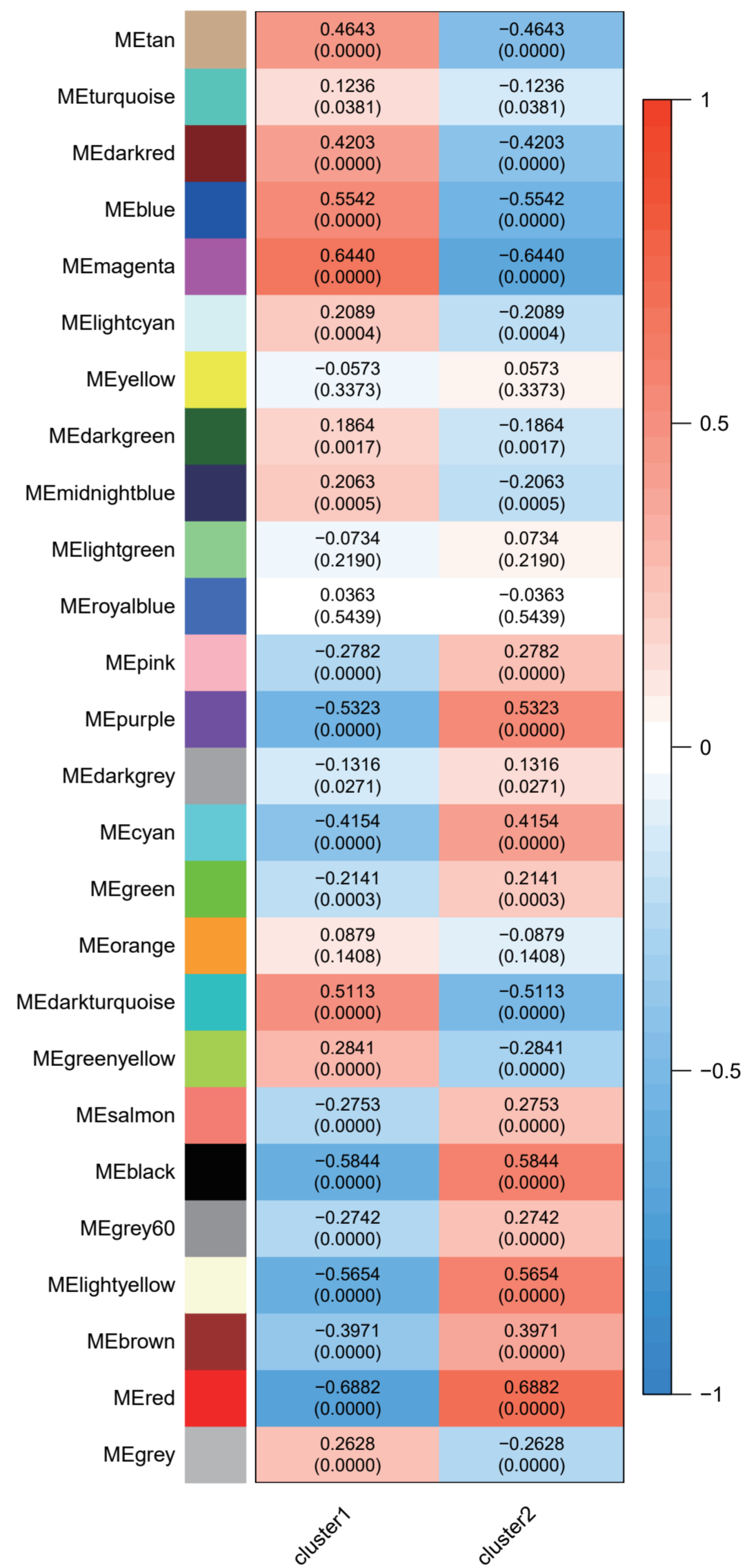

D

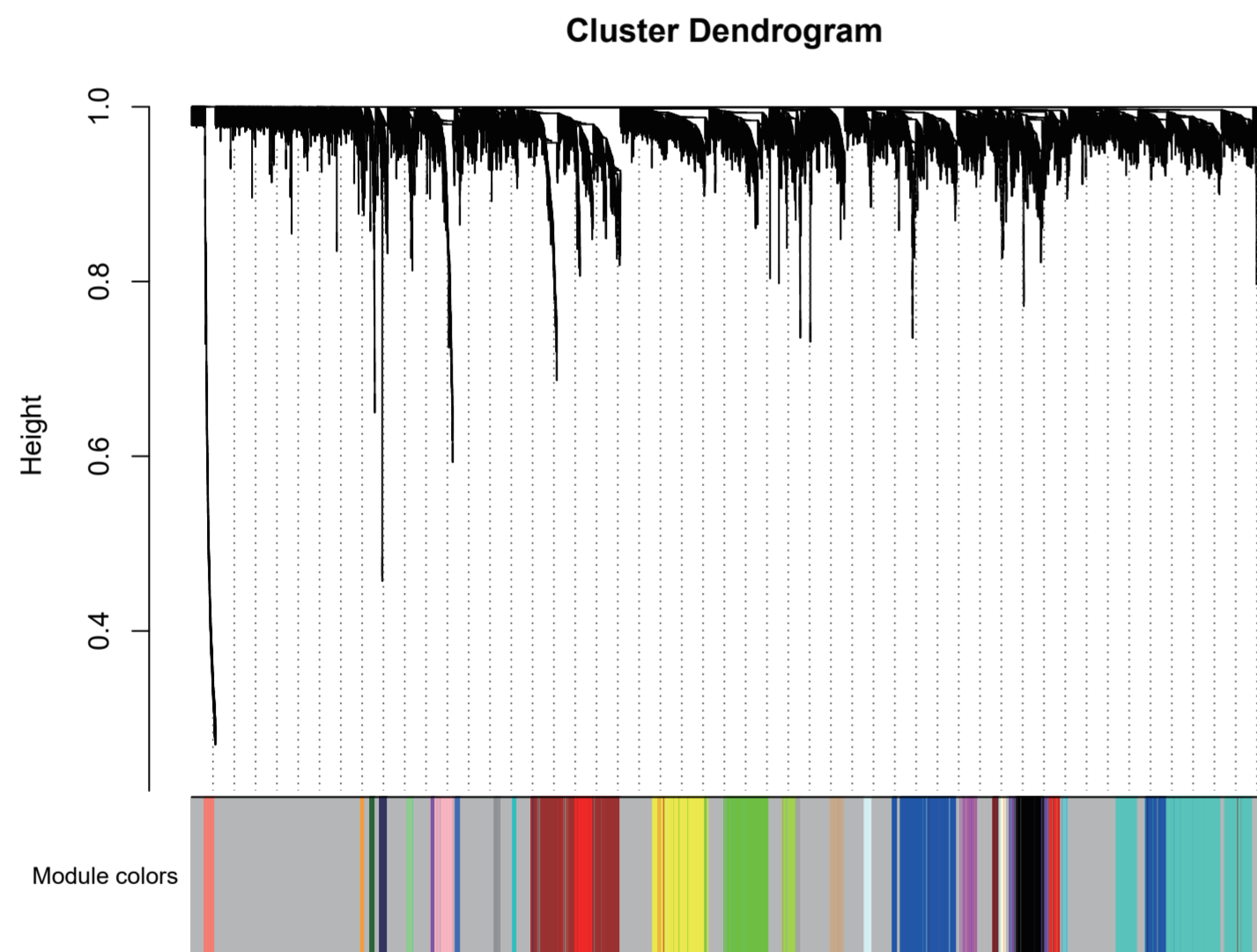

F

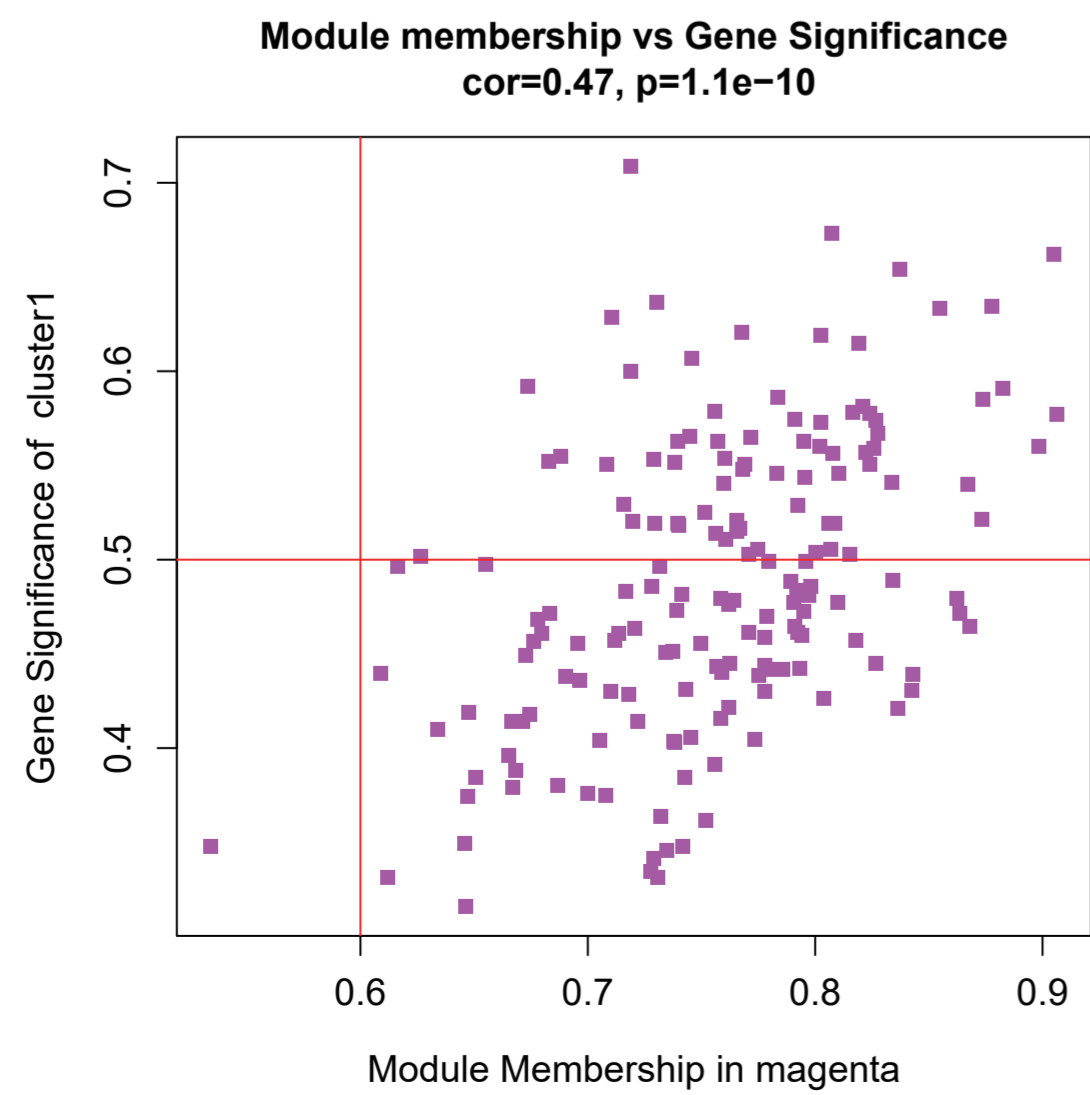

G

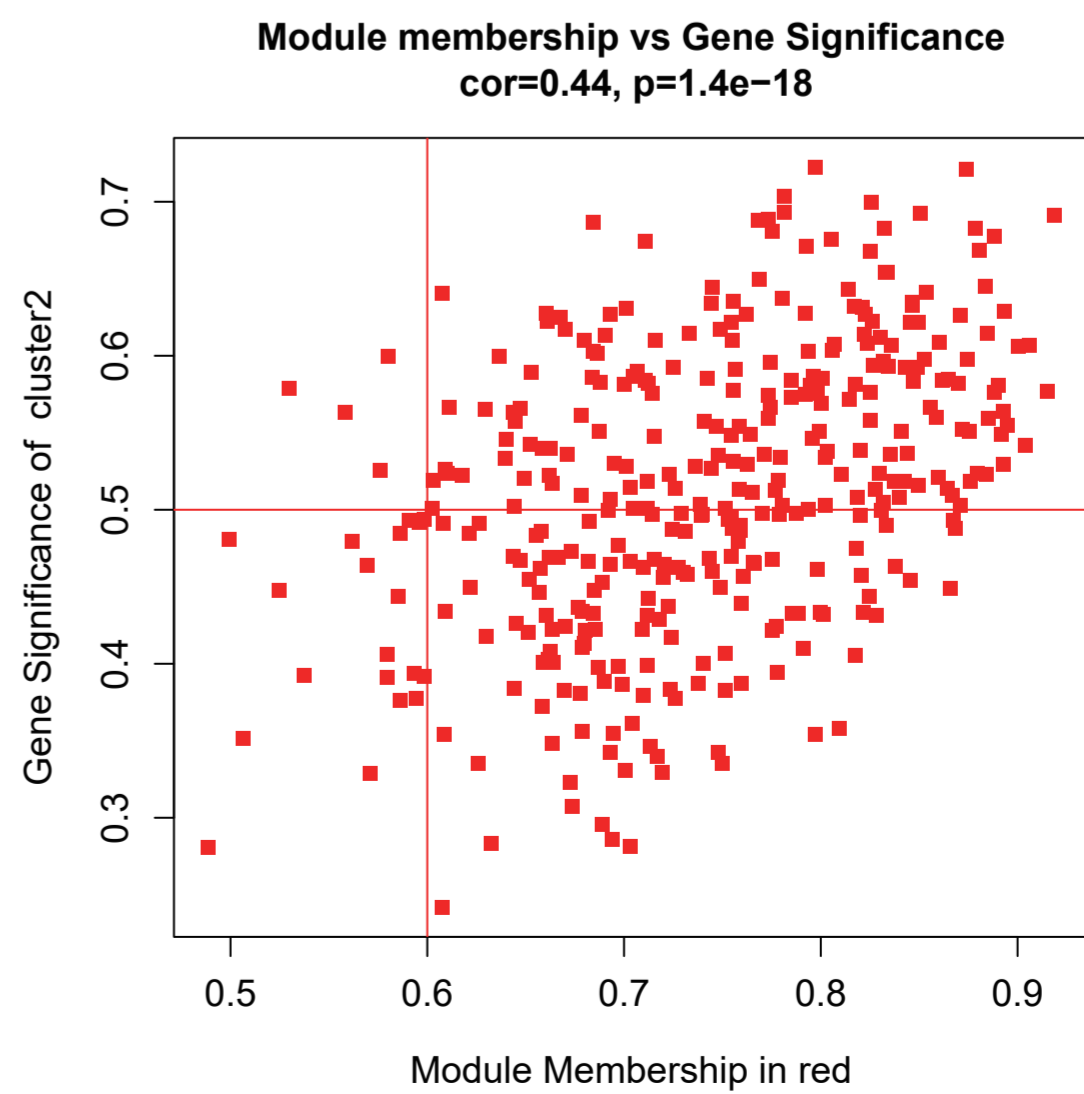

Supplement: Supplementary file 2 [file DataSheet2.PDF]

drug

gene

cluster

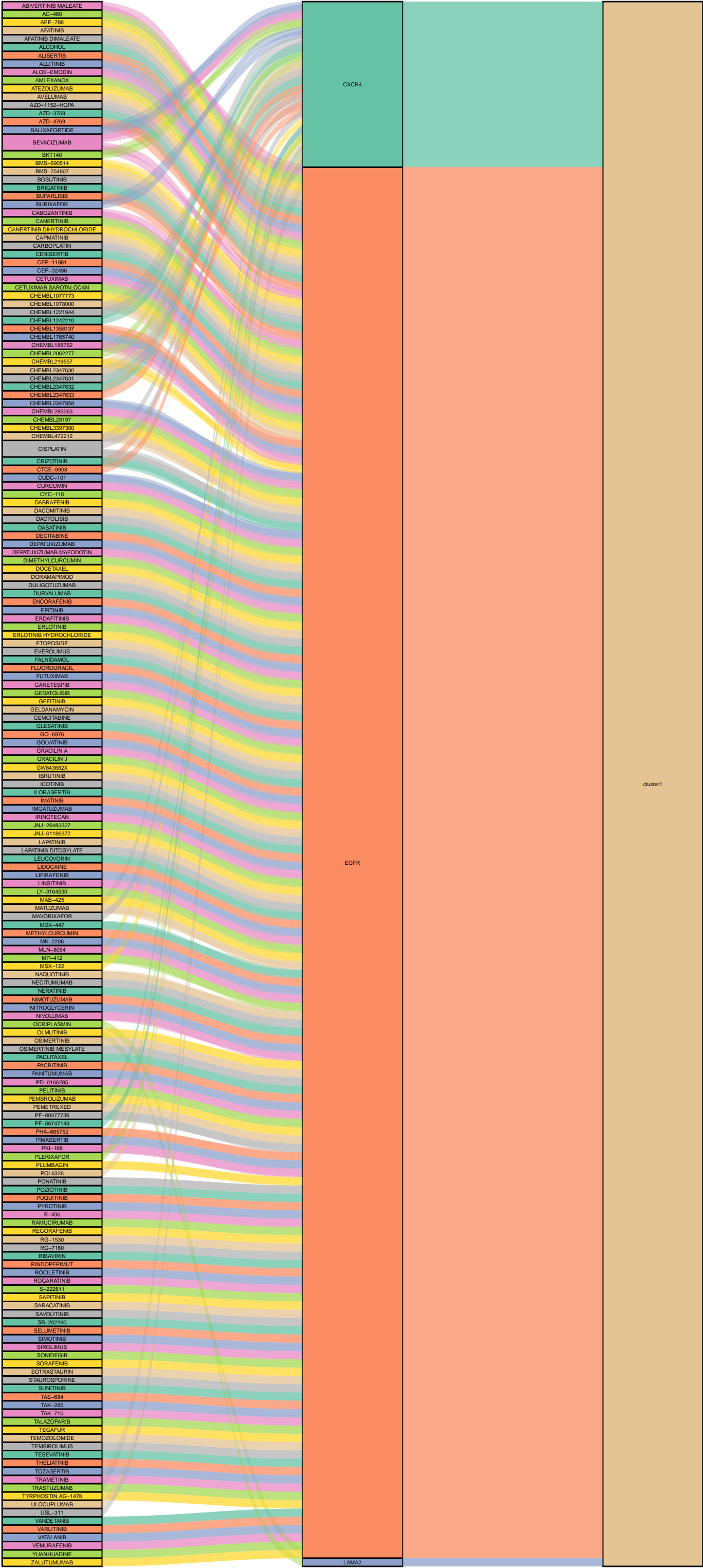

Supplement: Supplementary file 3 [file DataSheet4.PDF]

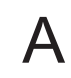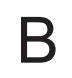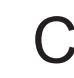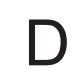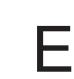

Supplement: Supplementary file 4 [file DataSheet6.PDF]

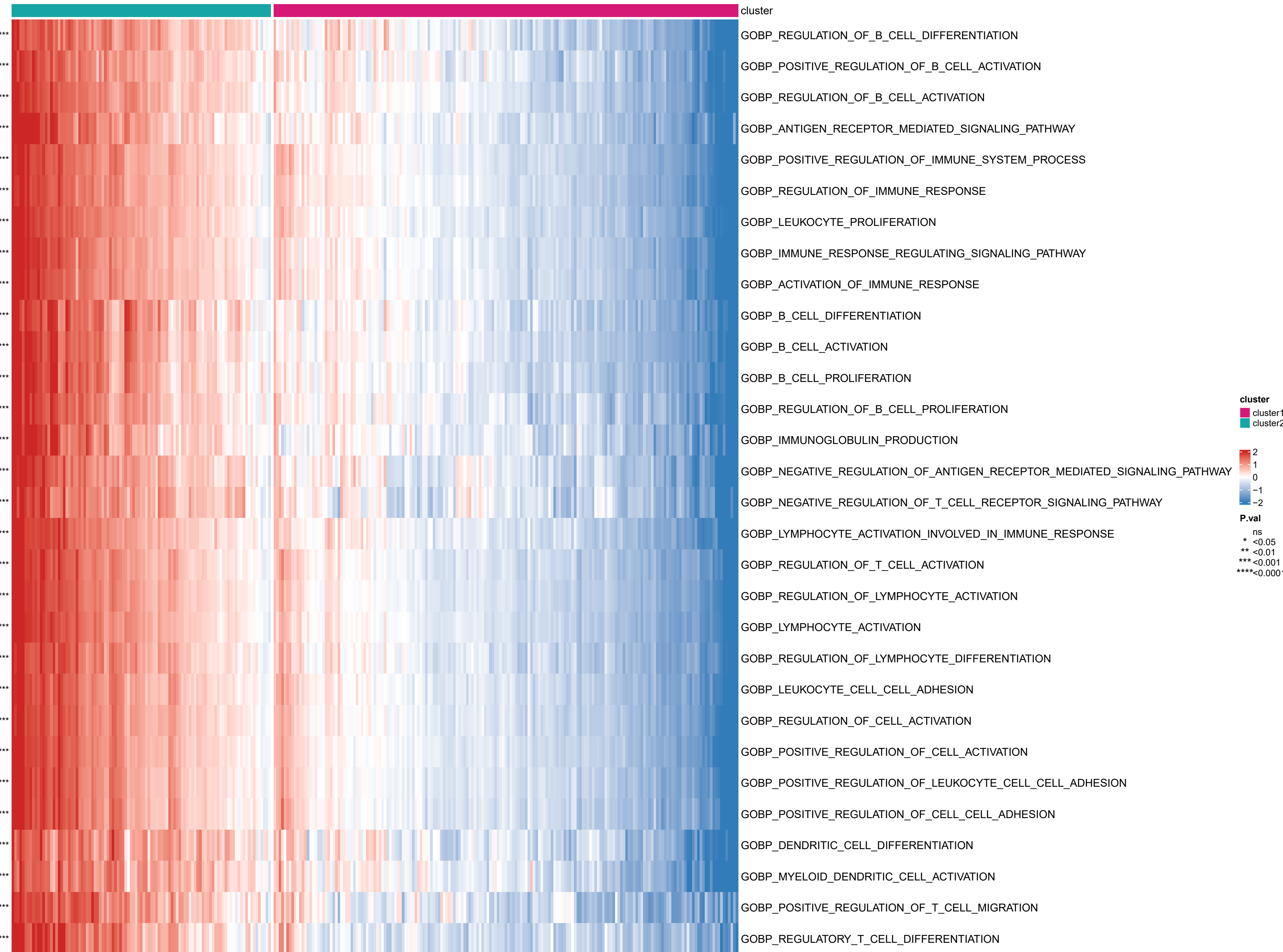

Supplement: Supplementary file 5 [file DataSheet9.PDF]

A

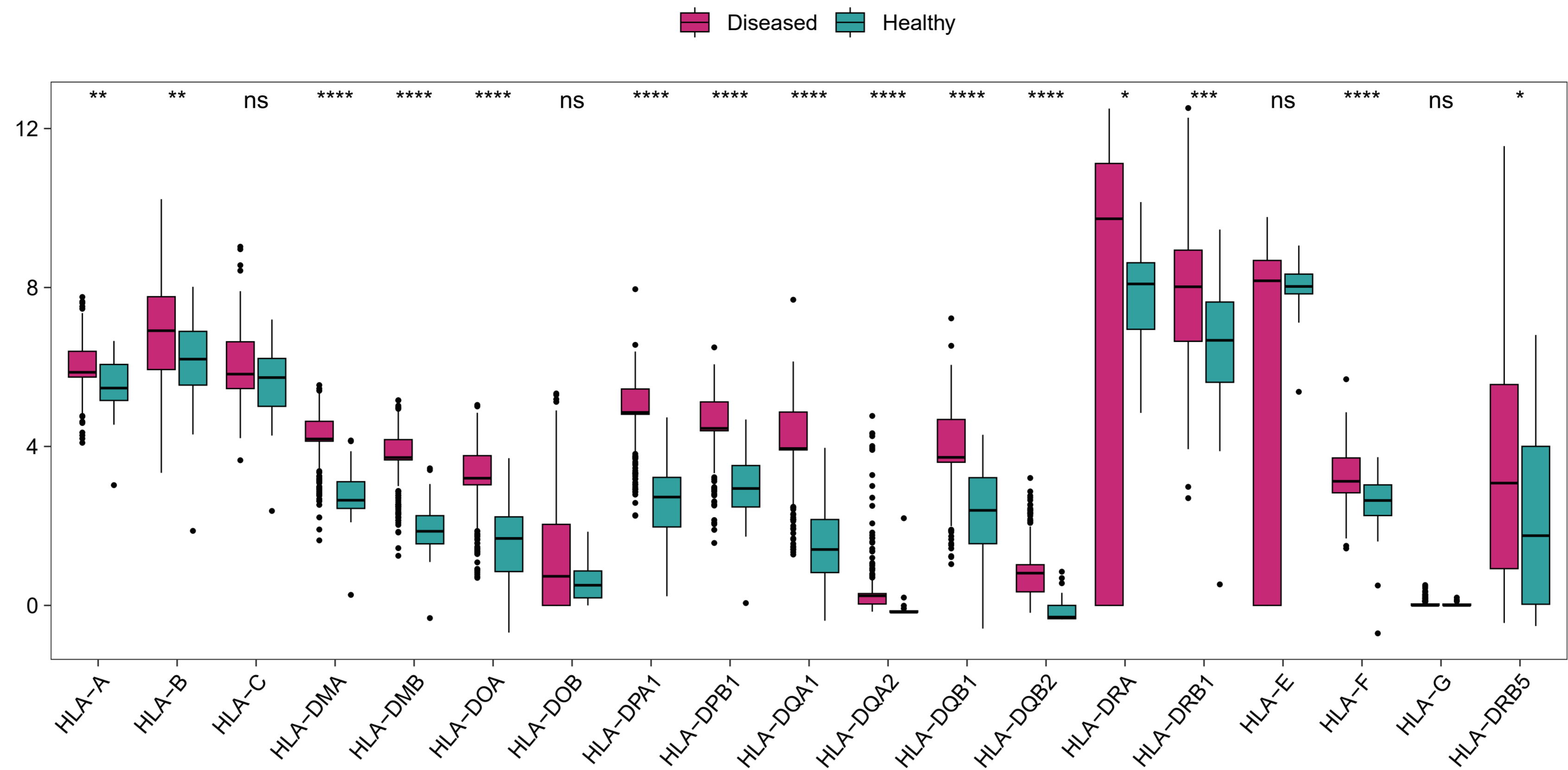

B

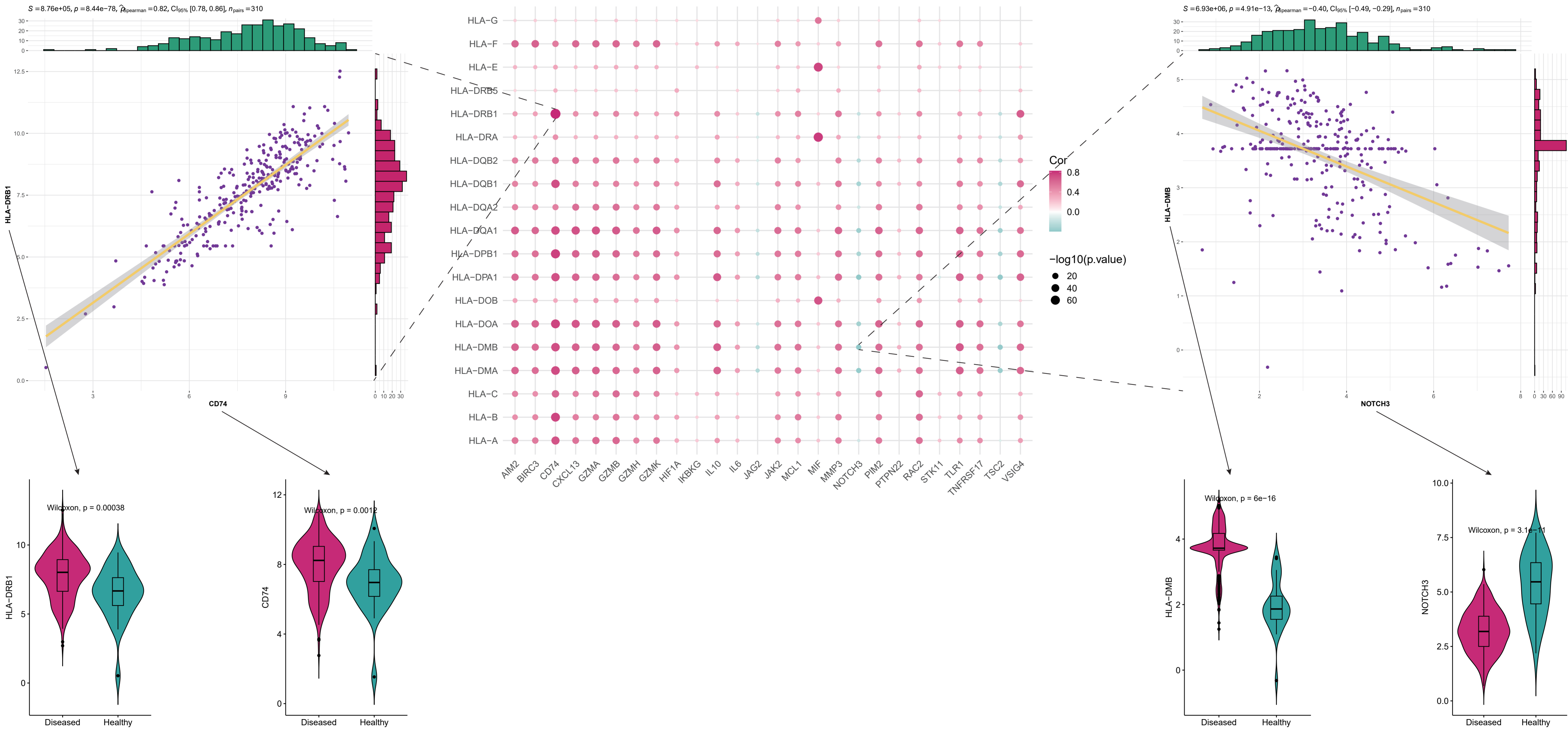

Supplement: Supplementary file 6 [file DataSheet11.PDF]

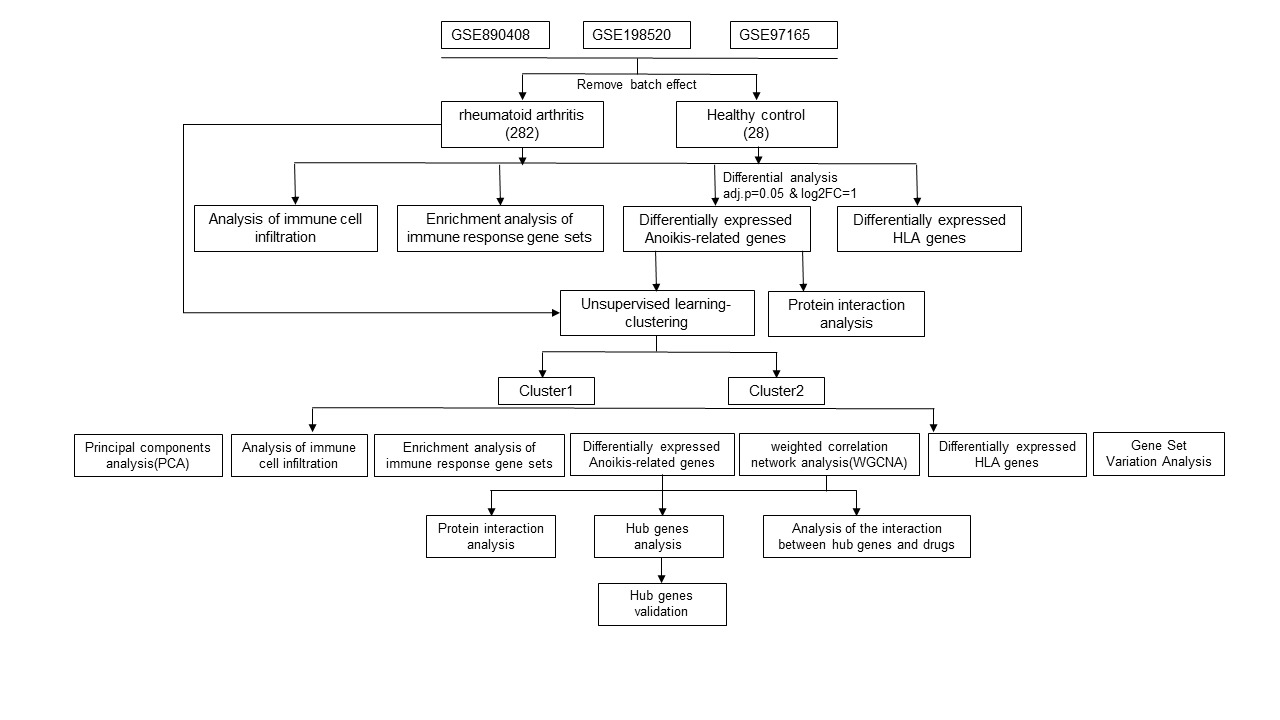

Supplement: Supplementary file 7 [file Image1.TIF]

A

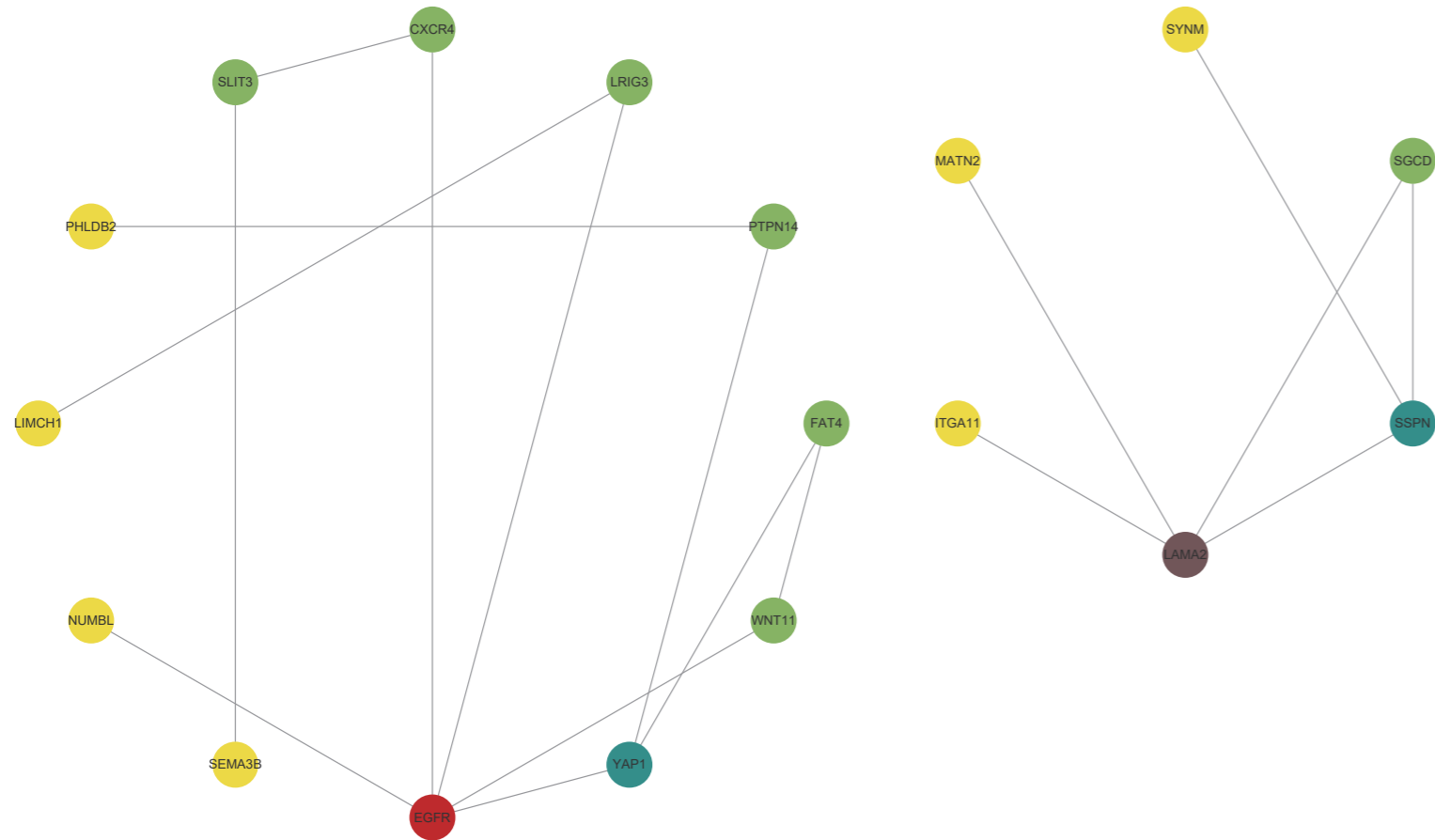

B

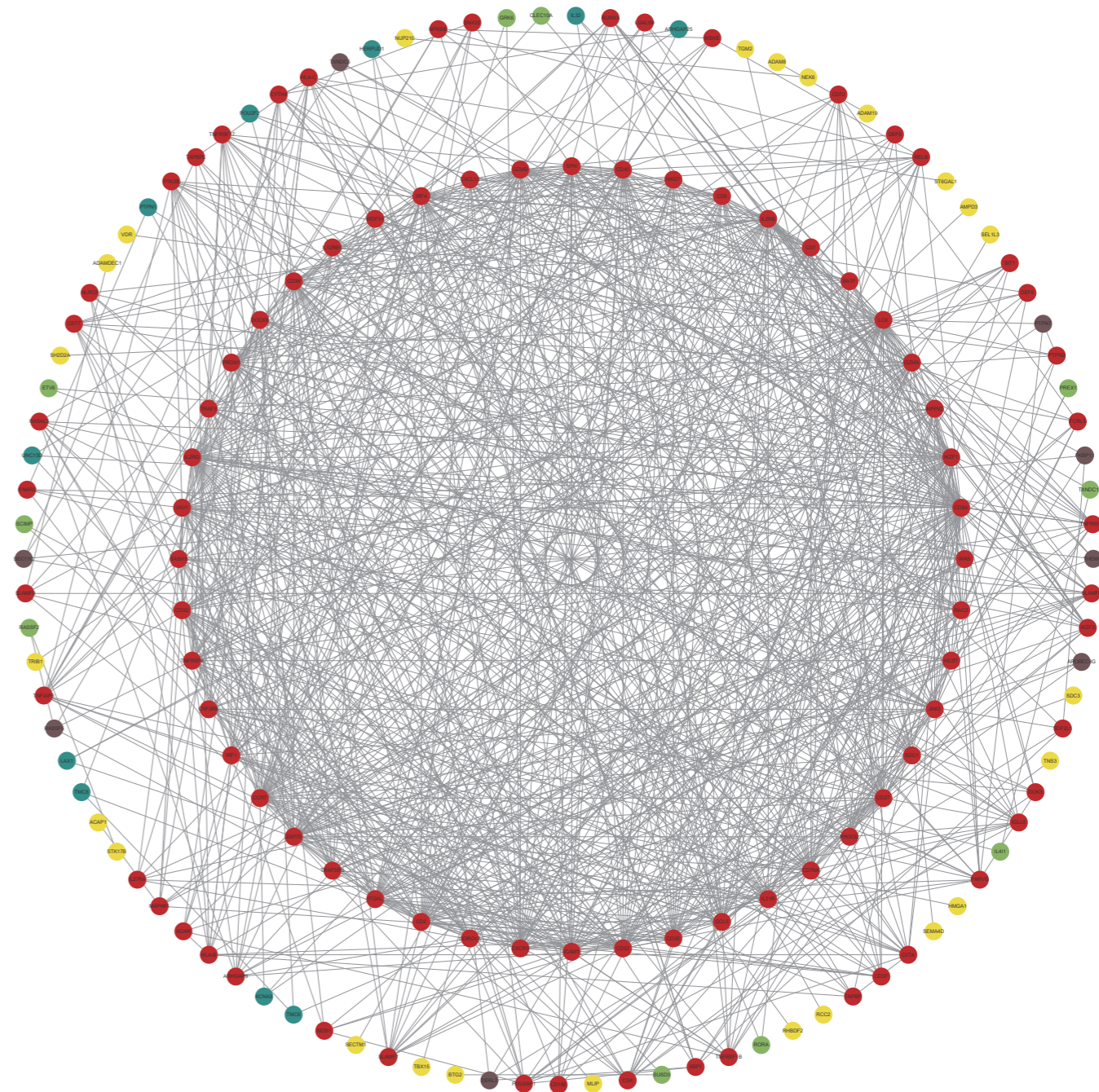

Supplement: Supplementary file 8 [file DataSheet3.PDF]

A

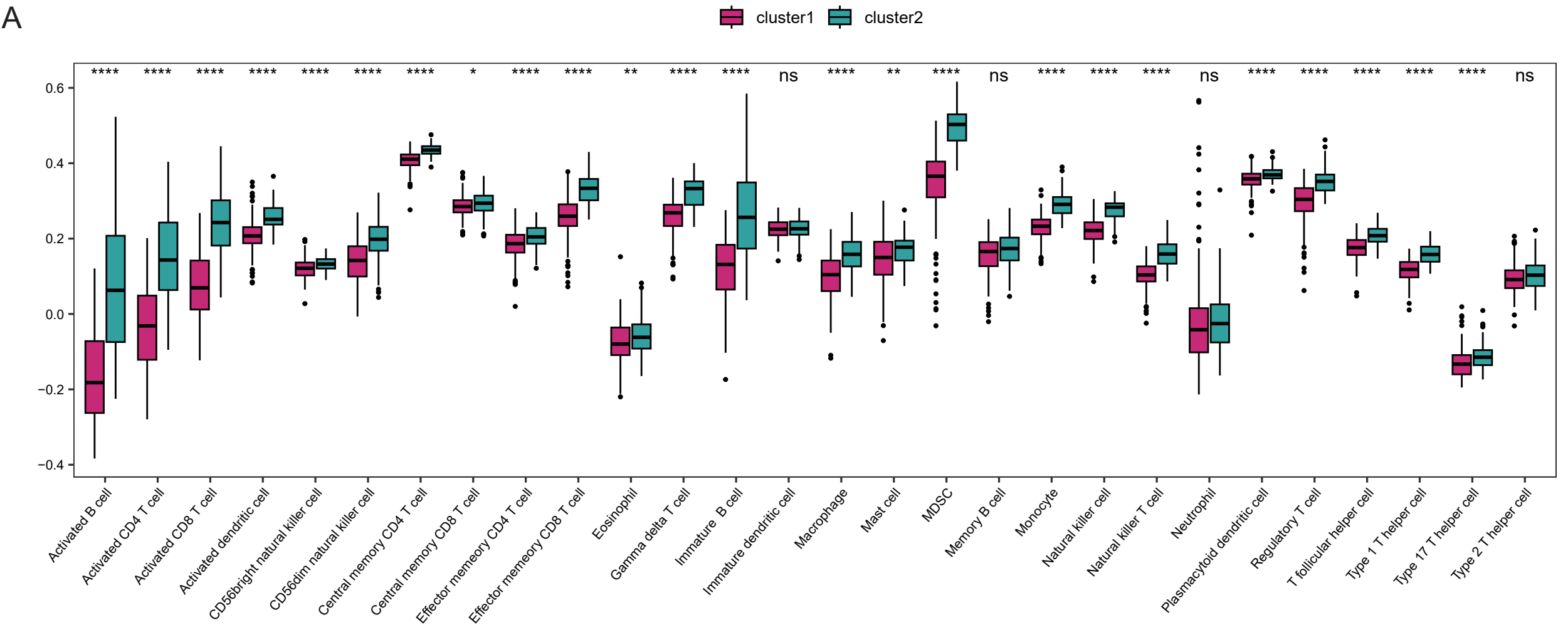

B

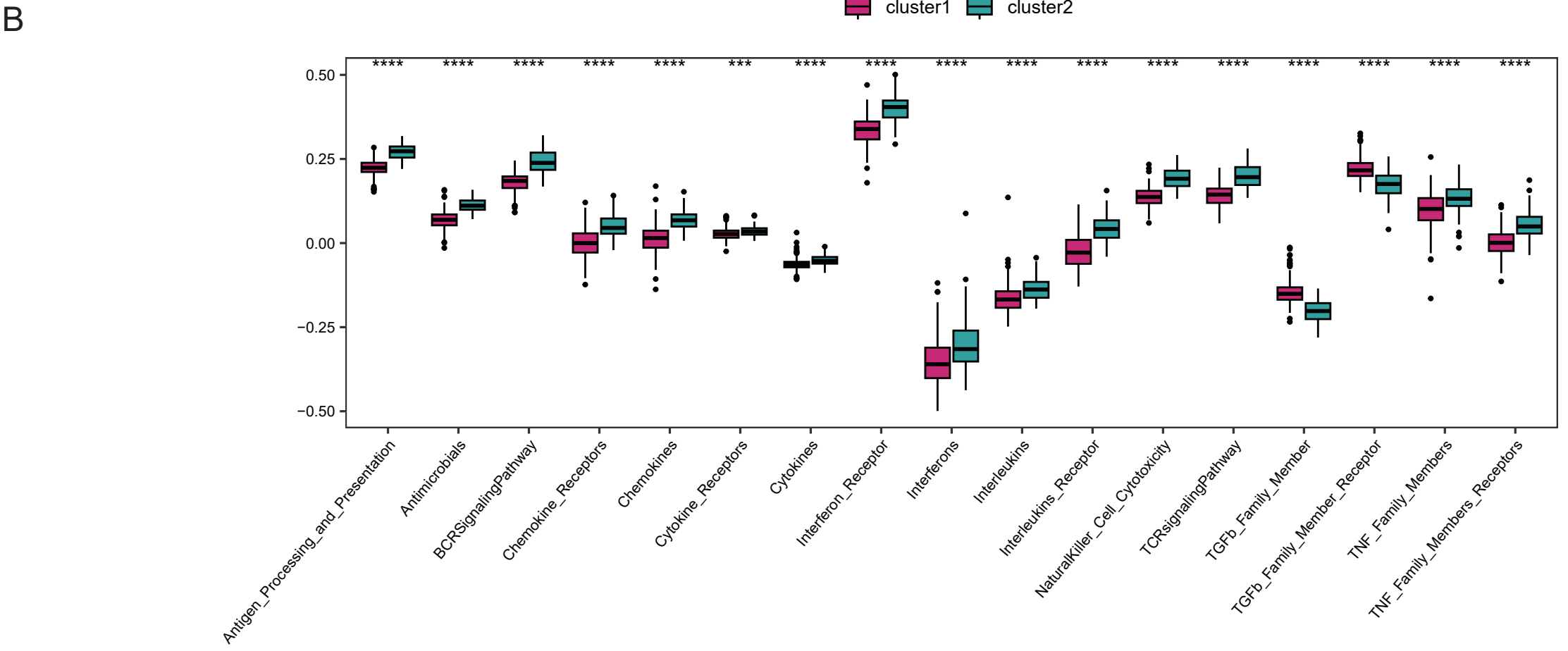

C

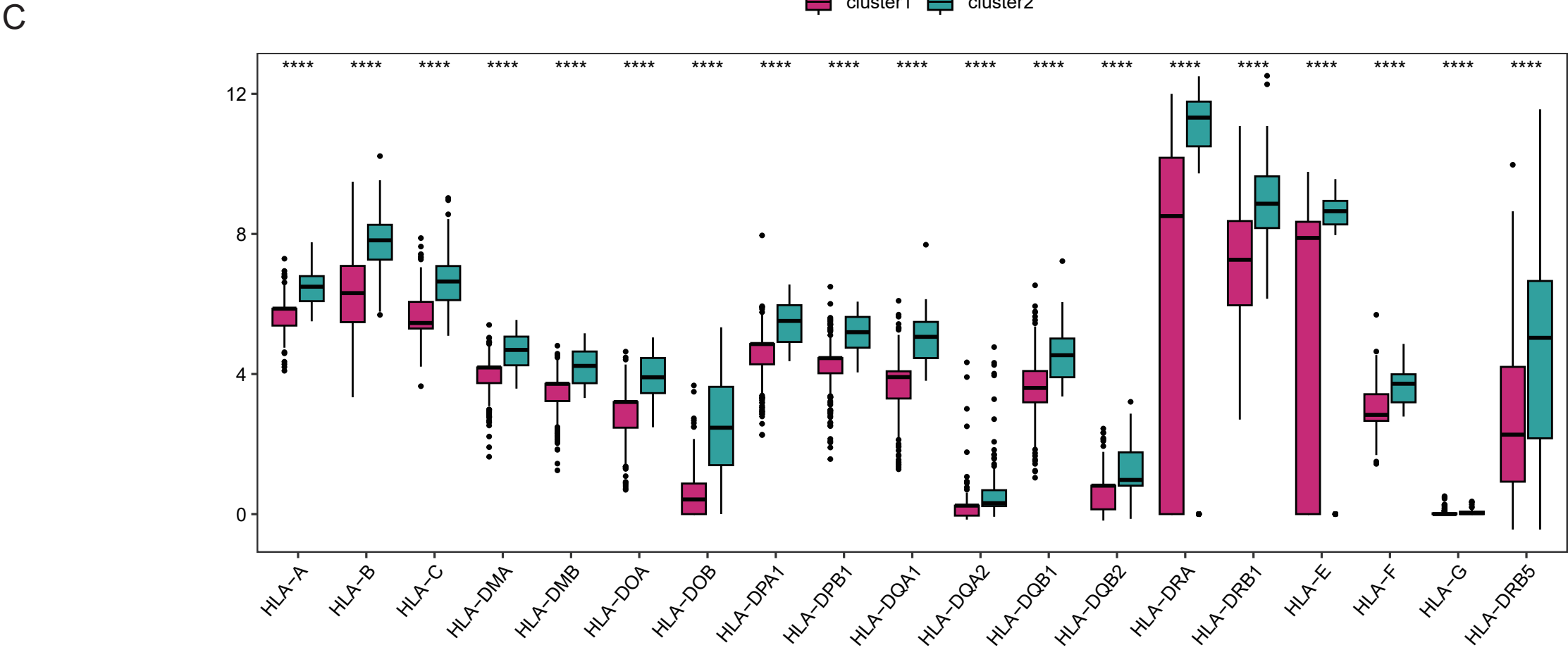

Supplement: Supplementary file 9 [file DataSheet1.PDF]

drug

gene

cluster

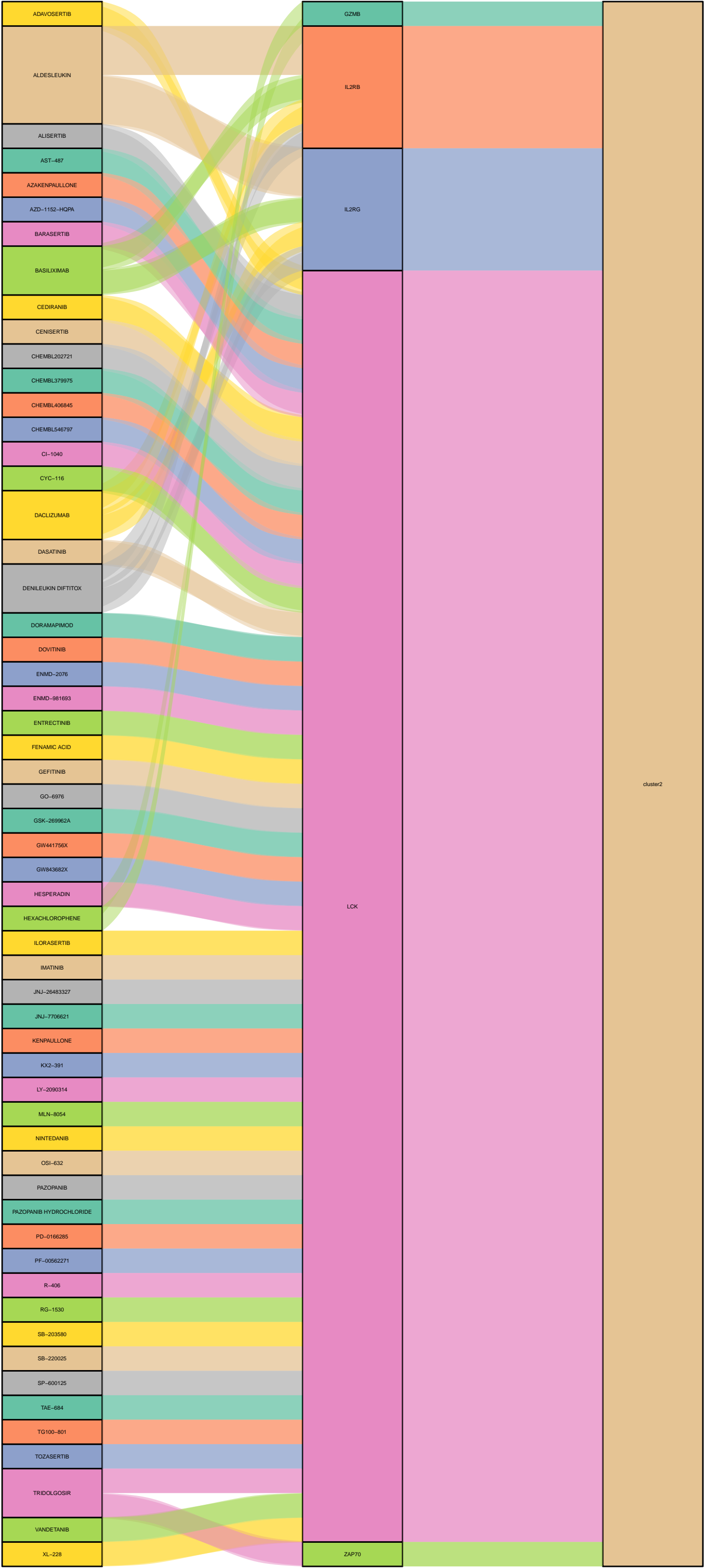

Supplement: Supplementary file 10 [file DataSheet5.PDF]

A

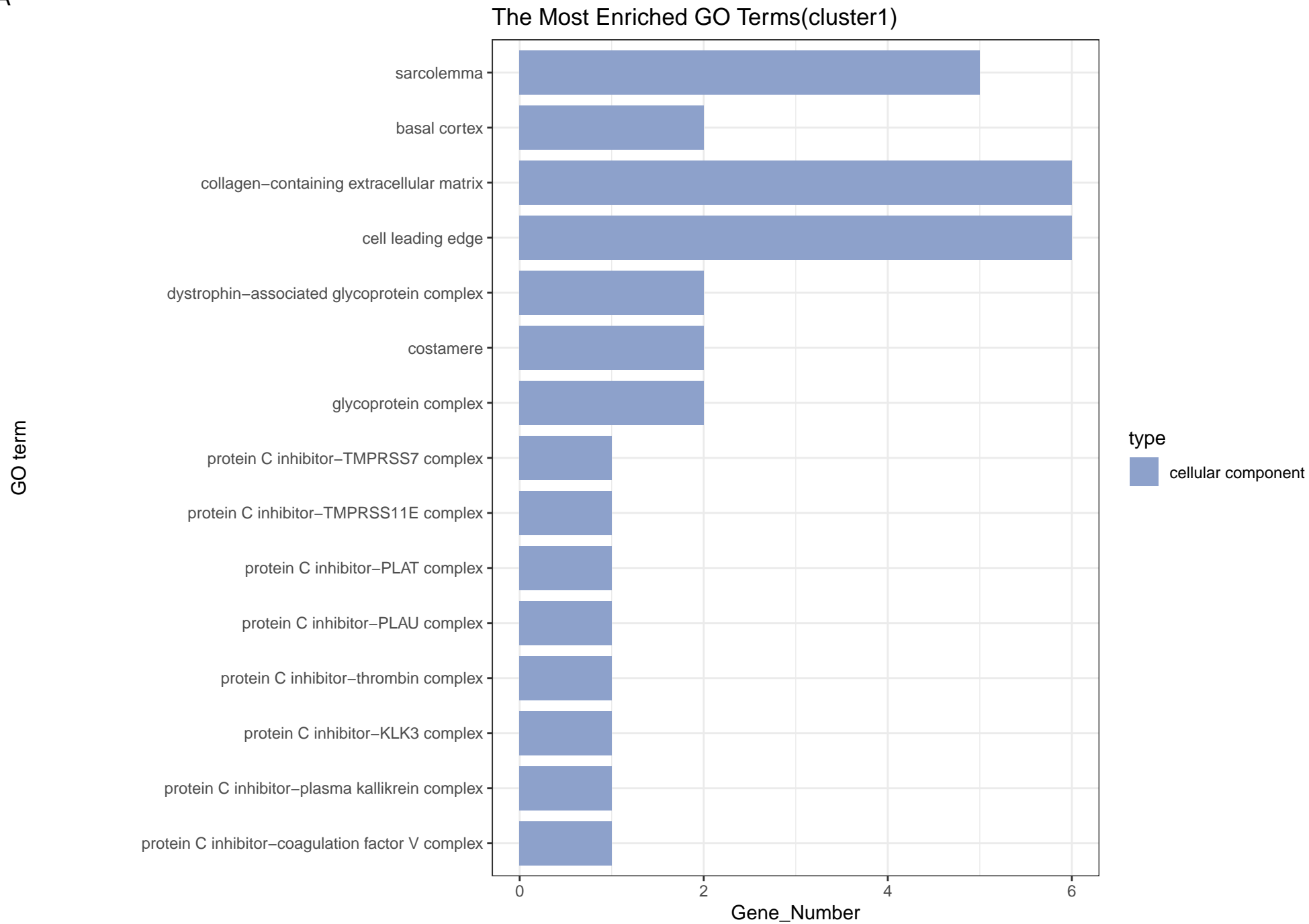

B

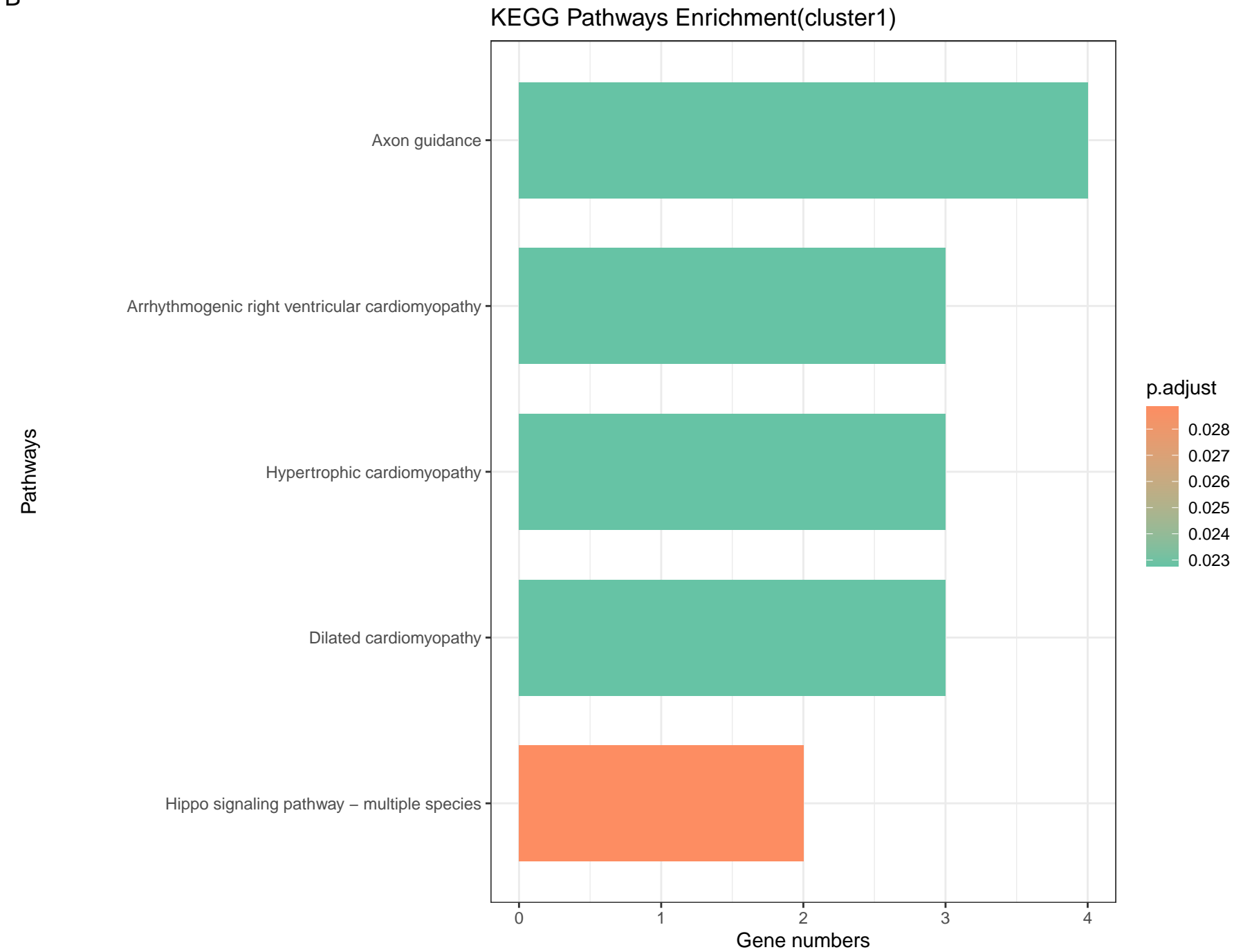

C

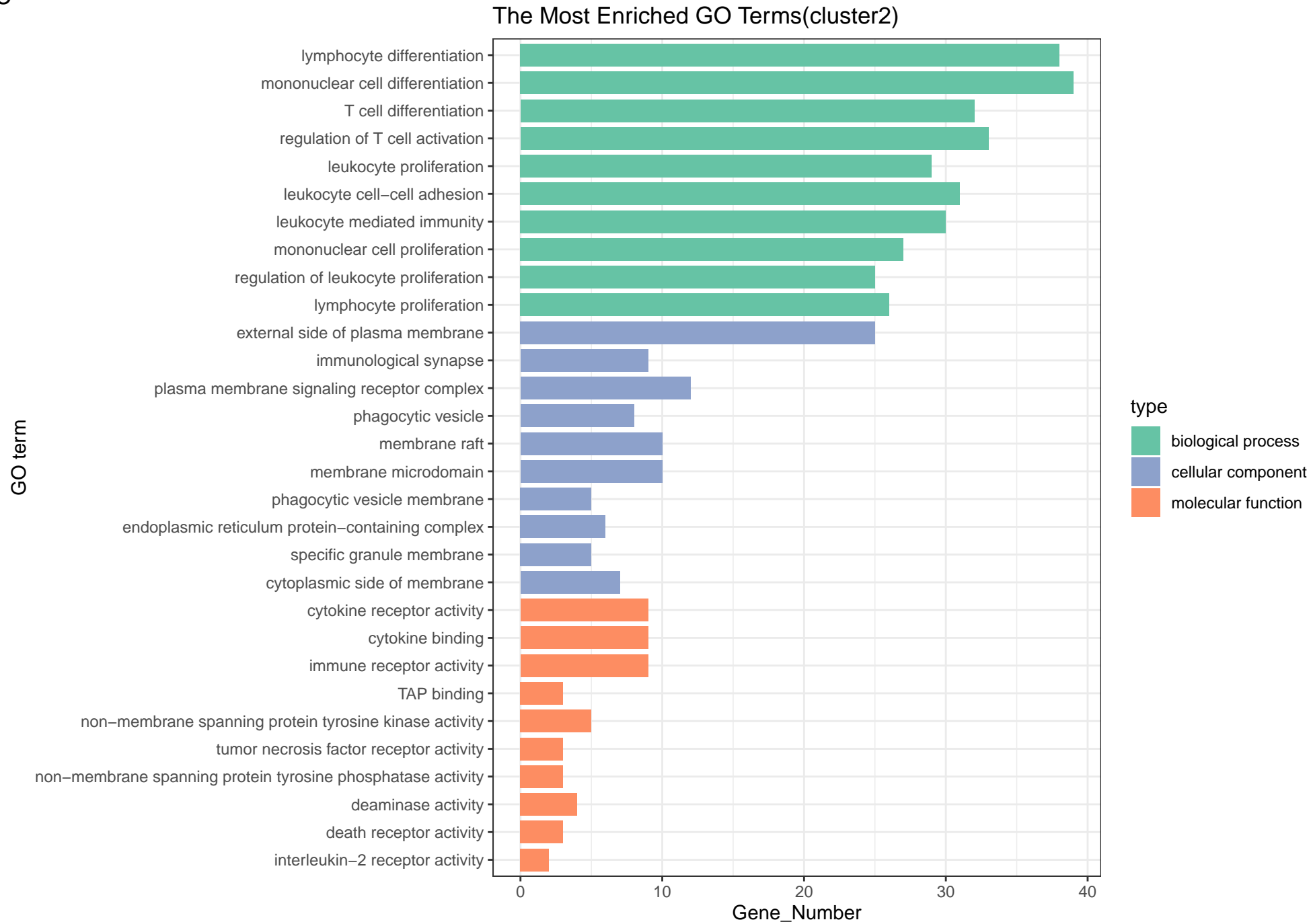

D

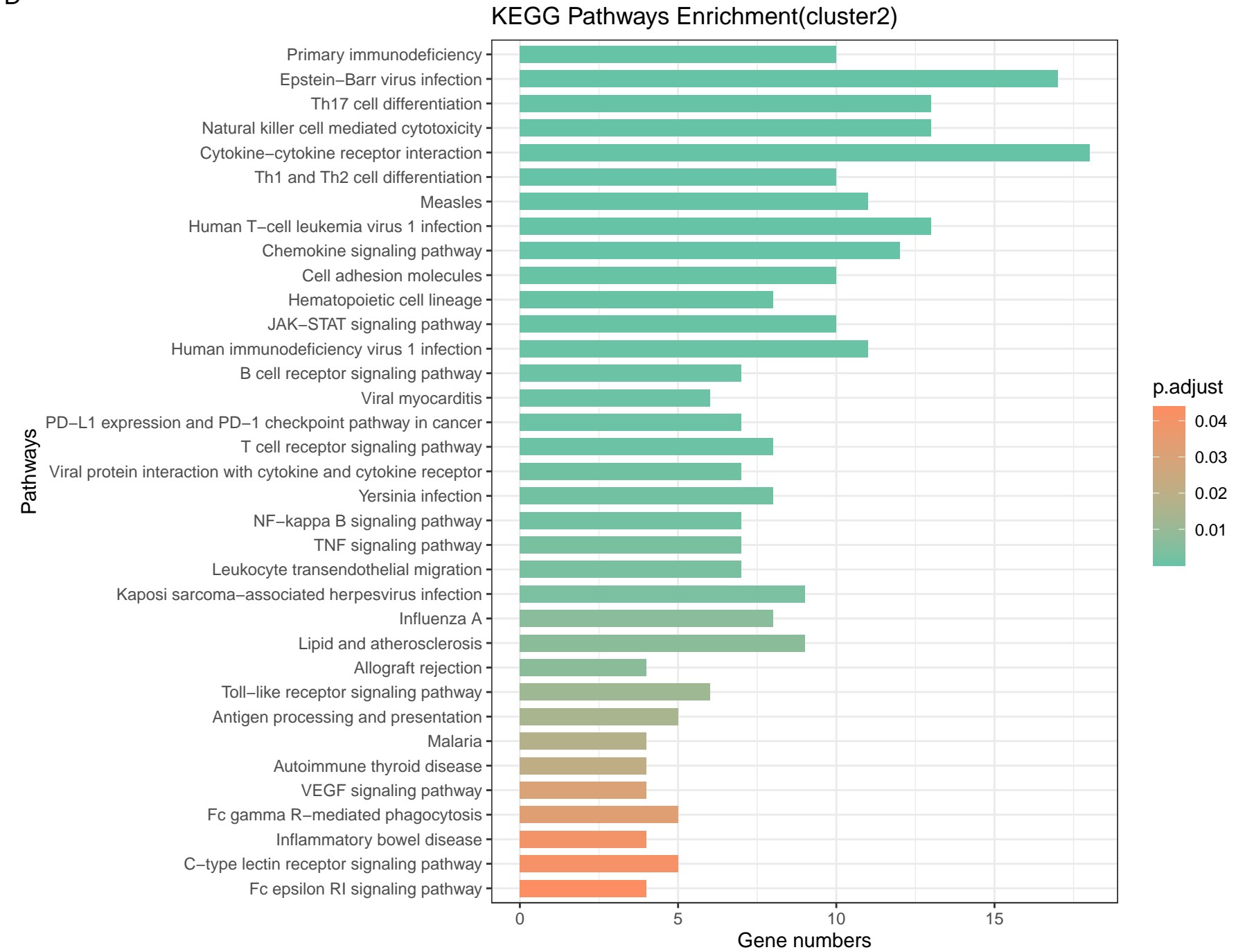

Supplement: Supplementary file 11 [file DataSheet12.PDF]

A

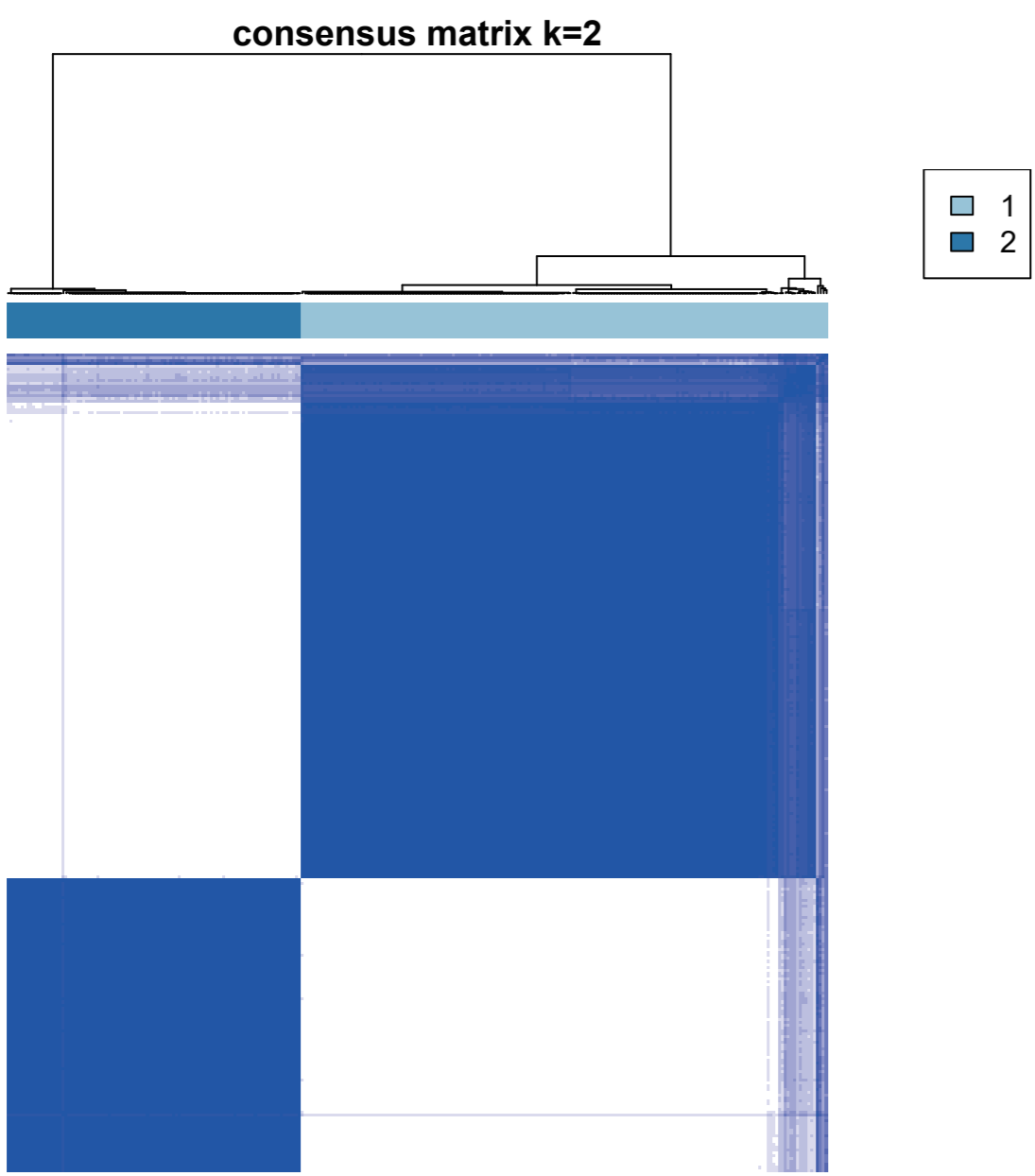

B

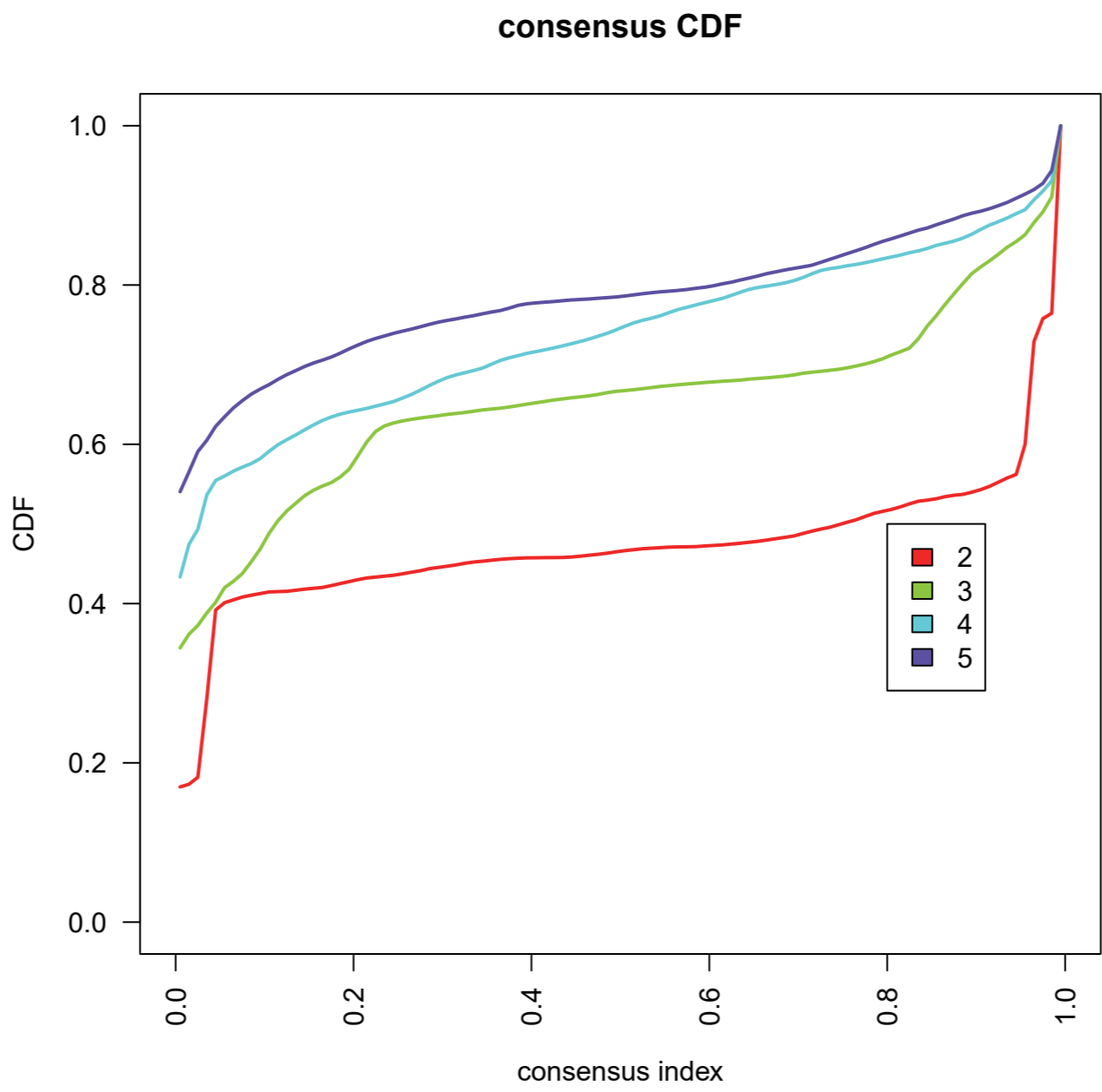

C

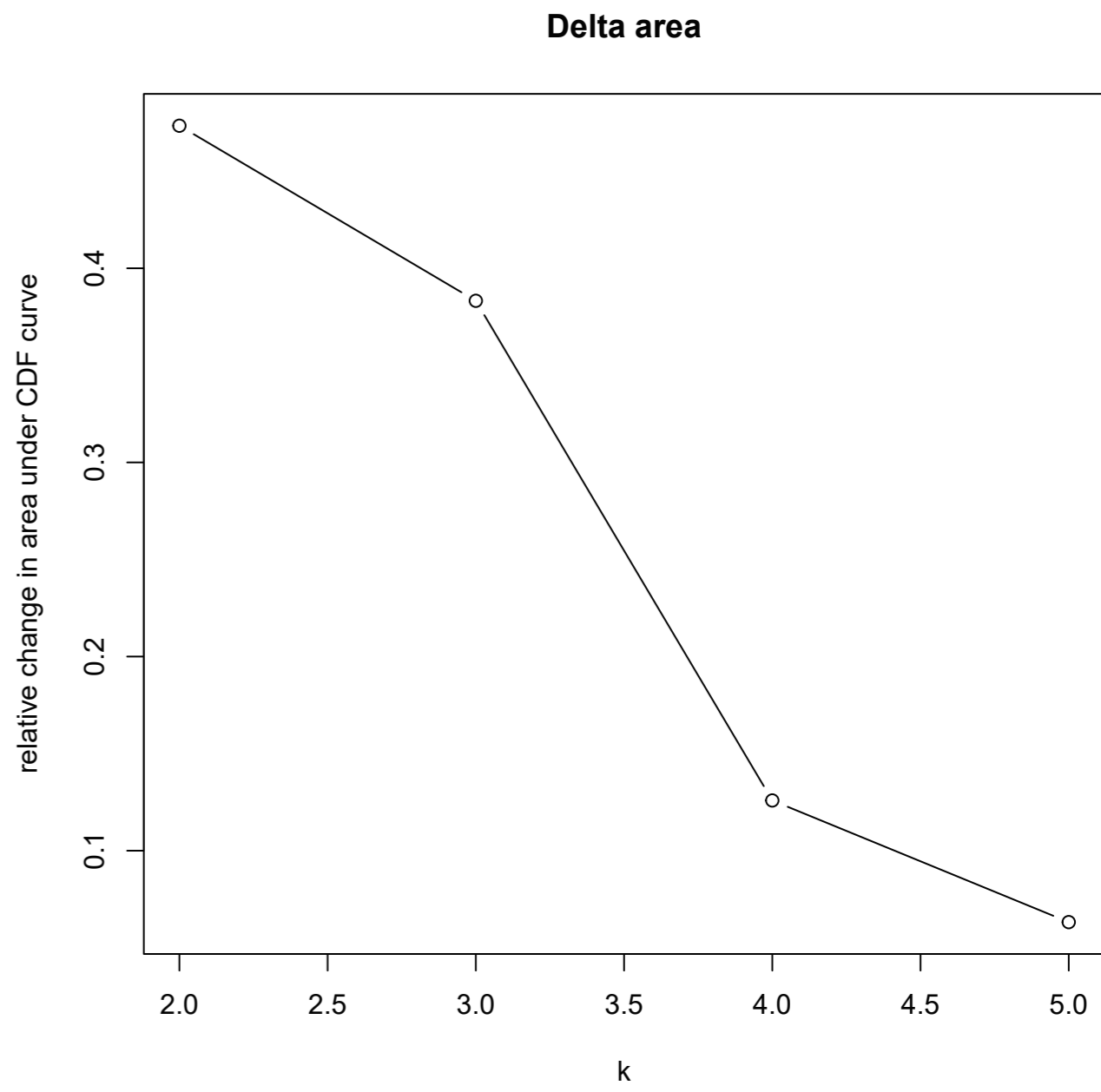

D

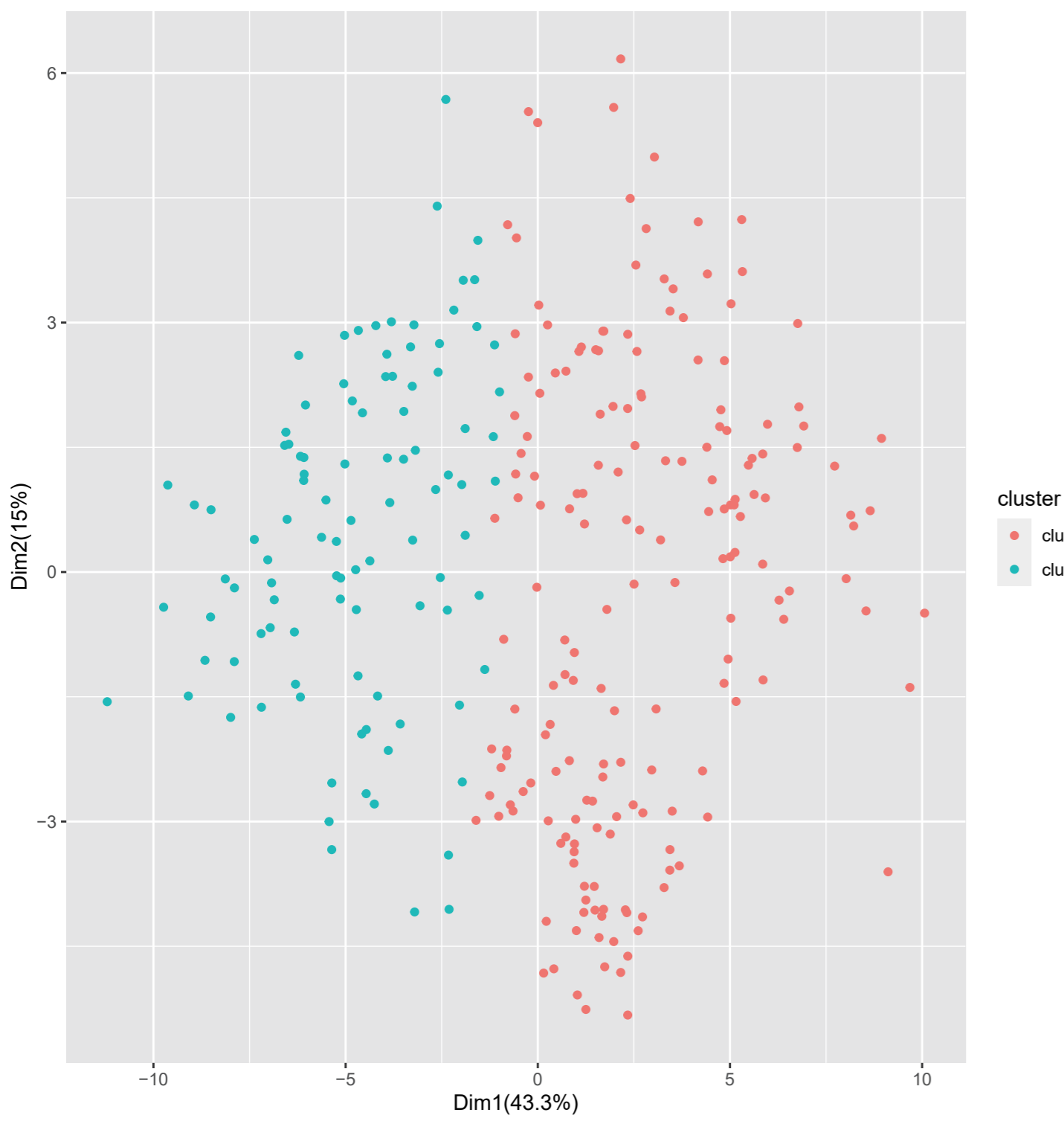

E

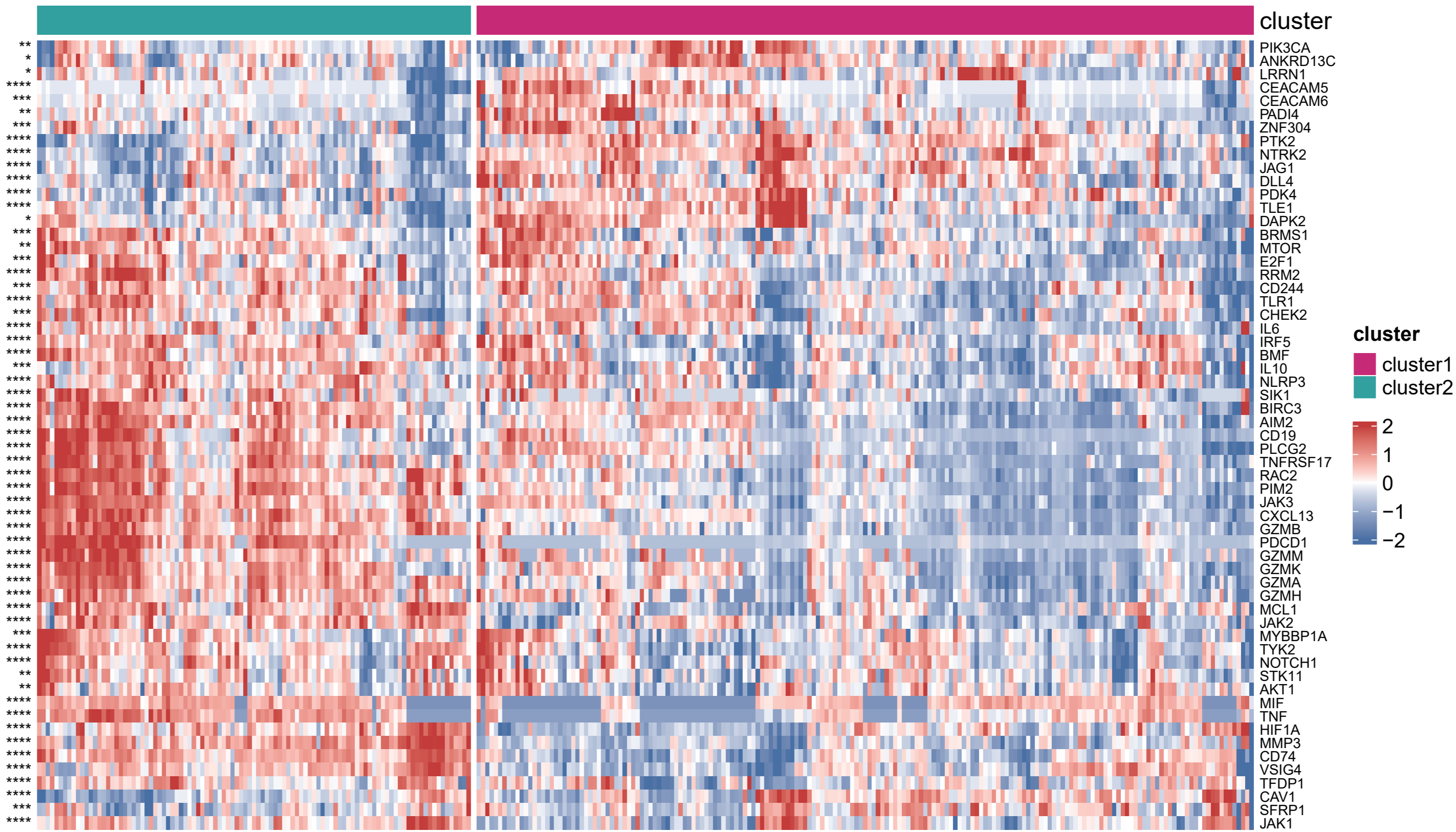

F

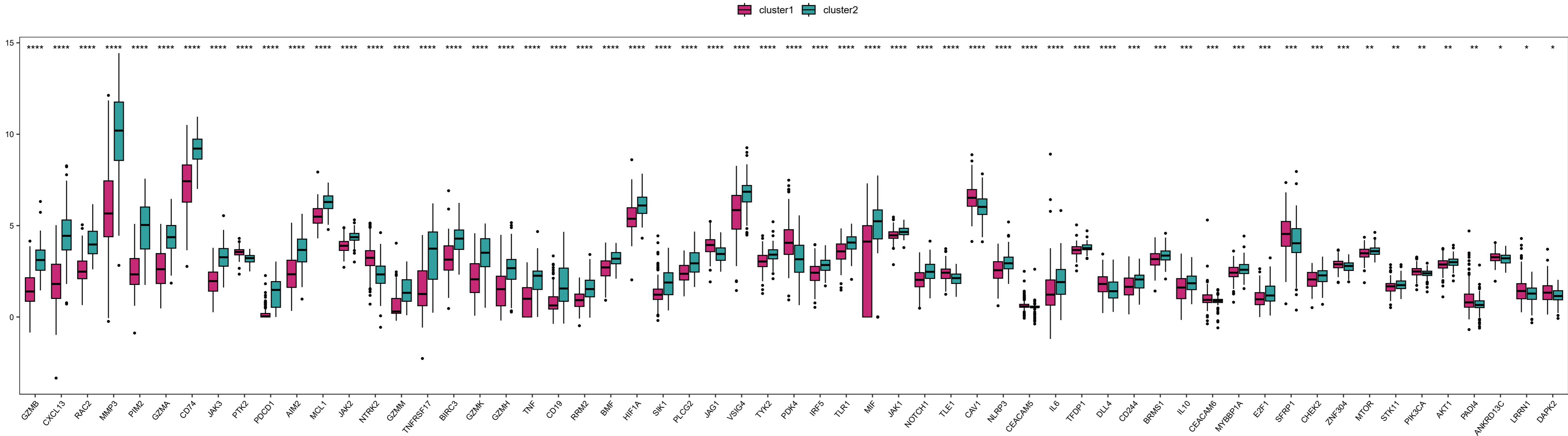

Supplement: Supplementary file 12 [file DataSheet8.PDF]

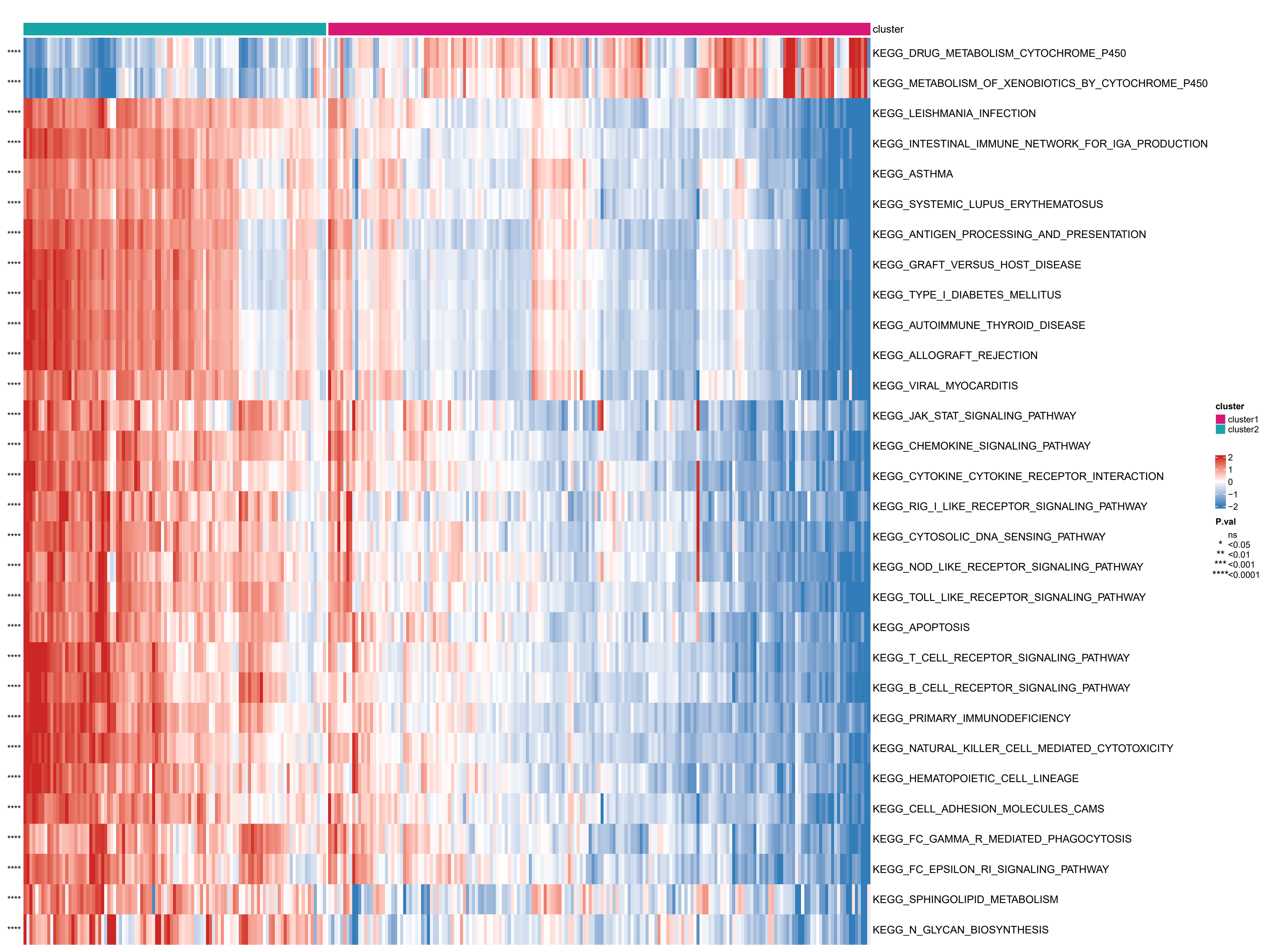

Supplement: Supplementary file 13 [file DataSheet10.PDF]
